# Genome-wide association studies identify OsWRKY53 as a key regulator of salt tolerance in rice

**DOI:** 10.1038/s41467-023-39167-0

**Published:** 2023-06-15

**Authors:** Jun Yu, Chengsong Zhu, Wei Xuan, Hongzhou An, Yunlu Tian, Baoxiang Wang, Wenchao Chi, Gaoming Chen, Yuwei Ge, Jin Li, Zhaoyang Dai, Yan Liu, Zhiguang Sun, Dayong Xu, Chunming Wang, Jianmin Wan

**Affiliations:** 1 State Key Laboratory of Crop Genetics and Germplasm Enhancement, Jiangsu Collaborative Innovation Centre for Modern Crop Production, Nanjing Agricultural University, Nanjing, 210095 China; 2 Southern Japonica Rice R&D Corporation Ltd, Key Laboratory of Biology, Genetics and Breeding of Japonica Rice in the Mid-lower Yangtze River, Ministry of Agriculture, Nanjing, 210095 China; 3 Department of Immunology, The University of Texas Southwestern Medical Centre, Dallas, TX 75390 USA; 4 MOA Key Laboratory of Plant Nutrition and Fertilization in Lower-Middle Reaches of the Yangtze River, Nanjing Agricultural University, Nanjing, 210095 China; 5 Lianyungang Academy of Agricultural Science, Lianyungang, Jiangsu 222006 China; 6 National Key Facility for Crop Gene Resources and Genetic Improvement, Institute of Crop Science, Chinese Academy of Agricultural Sciences, Beijing, 100081 China

**Keywords:** Plant breeding, Salt, Agricultural genetics, Quantitative trait

## Abstract

Salinity stress progressively reduces plant growth and productivity, while plant has developed complex signaling pathways to confront salt stress. However, only a few genetic variants have been identified to mediate salt tolerance in the major crop rice, and the molecular mechanism remains poorly understood. Here, we identify ten candidate genes associated with salt-tolerance (ST) traits by performing a genome-wide association analysis in rice landraces. We characterize two ST-related genes, encoding transcriptional factor OsWRKY53 and Mitogen-activated protein Kinase Kinase OsMKK10.2, that mediate root Na^+^ flux and Na^+^ homeostasis. We further find that OsWRKY53 acts as a negative modulator regulating expression of *OsMKK10.2* in promoting ion homeostasis. Furthermore, OsWRKY53 trans-represses *OsHKT1;5* (*high-affinity K*^*+*^ *transporter 1;5*), encoding a sodium transport protein in roots. We show that the OsWRKY53-OsMKK10.2 and OsWRKY53-OsHKT1;5 module coordinate defenses against ionic stress. The results shed light on the regulatory mechanisms underlying plant salt tolerance.

## Abstract (web summary)

Only a few genetic variants have been identified to mediate salt tolerance in major crops and their molecular mechanisms are largely unknown. Here, the authors identify WRKY53 as a negative regulator of salt tolerance in rice, and show that it directly trans-regulates expression of *MKK10.2* and *HKT1;5* to meditate salt tolerance.

## Introduction

Soil salinity is one of the most common abiotic stresses affecting crop growth and productivity, and has caused negative impacts on food security, water crisis, and sustainable agriculture^1^. Salt stress is likely to retard rice growth or kill rice, especially at the seedling stage, resulting in significant yield reduction^2^. Salt tolerance (ST) is known to be a complex quantitative trait which is controlled by multiple genes and involves various molecular and biological processes^3^. The identification of functional interaction among genes in salinity tolerance is still an active but also challenging area of research. So far, only three major ST-related genes *SKC1* (*HKT1;5*, *high-affinity K*^*+*^ *transporter 1;5*), *HST1* (*Hitomebore salt tolerant 1*), and *RST1* (*Rice Salt Tolerant 1*) were previously isolated through positional cloning in rice^4–6^. HKT1;5 mediates Na^+^ influx in parenchyma cells of roots in regulating K^+^/Na^+^ homeostasis under salt stress^4,7^, which is similar to the function of AtHKT1 in regulating Na^+^ distribution in roots, leaves, and xylems in Arabidopsis^8^. Up to now, the detailed regulatory mechanisms of *OsHKT1;5* in ST remain obscure^9^. Meanwhile, the main determinants and the genetic mechanisms of ST have largely remained uncharacterized in rice, leading to limited progress in developing salt-tolerant rice cultivars.

Compared to the traditional positional cloning strategy of salt tolerance related genes^4,10^, which typically aims at one target gene, genome-wide association studies (GWAS) have the advantage of being able to identify multiple loci harboring association variants contributing to agronomic traits. However, the identified loci typically contain multiple genes needed to prioritize GWAS variants and further identify causal genes^11,12^. Previously, we designed a GWAS-based strategy to explore and prioritize candidate genes underlying NUE (Nitrogen Use Efficiency)-related traits followed by complementation validation experiments^12,13^.

The MPK cascades in stress signaling are present in organisms ranging from metazoans to fungi and plants^14^. MPK-related cascades are involved in ionic, osmotic, and oxidative stress signaling in plants. AtMPK6 is activated by phosphatidic acid and plasma membrane Na^+^/H^+^ antiporter (SOS1) is identified as a downstream target of AtMPK6^15^. However, the specific regulatory mechanisms of MPK cascades in salt stress signaling are still unclear.

We herein perform GWAS on ST-related traits and identify ten candidate genes, including *OsHKT1;5*, *OsWRKY53*, and *OsMKK10.2*. We demonstrate the functional interactions among these identified genes. Our results reveal that OsWRKY53 acts as a negative regulator of ST, and directly trans-regulates expressions of *OsMKK10.2* and *OsHKT1;5*. Our OsWRKY53-regulated network establishes a connection between upstream regulator and OsMPK cascades that is critical to the protection of rice from salinity stress. Our study highlights a central role of OsWRKY53 in transcriptional regulation of salt tolerance in rice.

## Results

### Identification and prioritization of salt tolerance-associated genes using GWAS and genome-wide selection

**Fig. 1:**
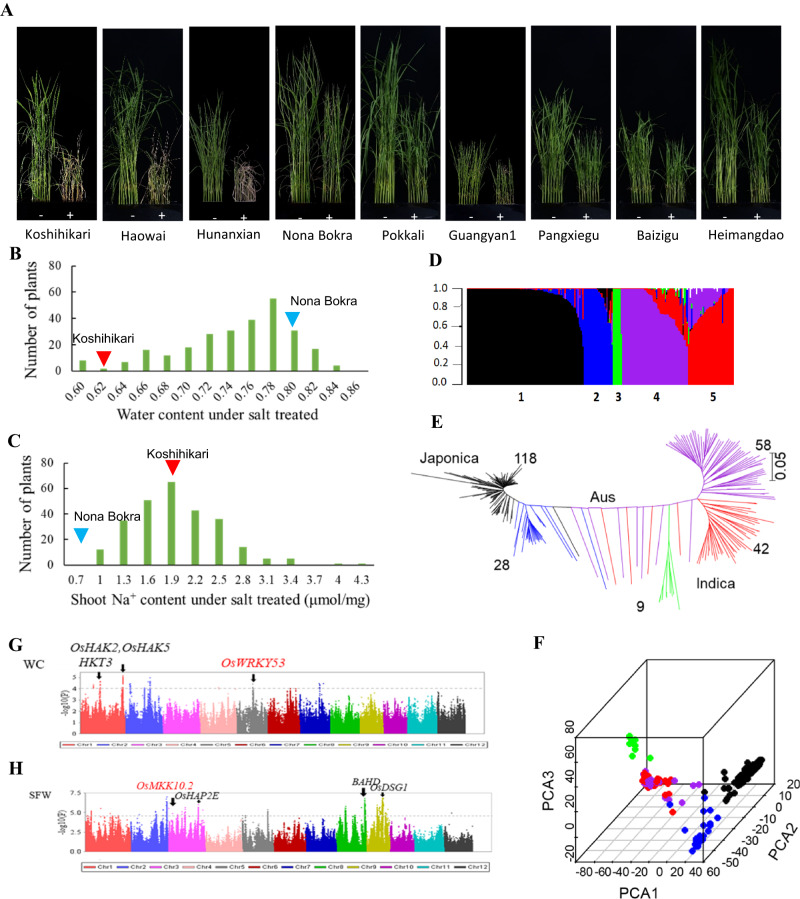
GWAS for the eight salt-tolerance traits. **A** Salt response of representative rice varieties in the GWAS. ^–^ and ^+^ represented control and 140 mM NaCl treatment, respectively. **B** Frequency distribution of water content under salt treated. Arrows represented control accessions, and Nona Bokra was tolerant to salt stress and Koshihikari was a salt sensitive variety. **C** Frequency distribution of shoot Na^+^ content under salt treated. **D** Population structure. The colored subsections within each vertical bar indicated membership coefficient (Q) of the accession to different clusters. **E** Neighbor-joining clustering of landraces based on genetic distance. The scale bar showed substitutions per site. The colors of the bar and the tree branch indicated the five groups identified through the STRUCTURE program. **F** Principal components analysis for the 268 rice varieties based on whole-genome sequence data. PC1, PC2, and PC3 indicated eigenvectors of top principal components 1, 2 and 3, respectively. **G** Overview of MLM with *OsWRKY53* highlighted in red. Scale: −log_10_ of *P* value of markers. Manhattan plots for WC. **H** Overview of MLM with *OsMKK10.2* highlighted in red. Scale: −log_10_ of *P* value of markers. Manhattan plots for SFW. Mixed linear model was used to calculate *P* value.

We assessed ST-related traits in a diverse rice population with 268 accessions, including 100 *indica*, 50 *aus*, and 118 *japonica* originating from around the world (Fig. 1A–F, Supplementary Figs. 1–3, Supplementary Table 1, Supplementary Data 1 and 2). We measured eight ST-related traits with the potential to improve rice salt tolerance, including shoot height (SH), shoot fresh weight (SFW), shoot dry weight (SDW), shoot water content (SWC), shoot Na^+^ concentration (SNC), shoot K^+^ concentration (SKC), shoot Na^+^/K^+^ concentration ratio (SNKR), and survival rate (SR) (Supplementary Fig. 1) which was positively correlated with SH, SFW, SDW, SWC, SNC and negatively correlated with SNC, SNKR, and SKC (Supplementary Table 2, see Supplementary Note 1).

To identify factors regulating salt tolerance in rice, we carried out GWAS using 2,535,266 imputed single nucleotide variants (SNVs) generated by genotyping by sequencing (GBS) (see Supplementary Note 2). A total of 83 associated loci associated with at least one of the eight salt-tolerance-related traits were identified (Fig. 1G, H, Supplementary Fig. 3c, Supplementary Data 3). Through evaluation for their likely function based on publicly available genomic annotations (Supplementary Fig. 4, Supplementary Table 3 and Supplementary Data 3), 19 associated loci that contained at least one gene within the locus were identified (Supplementary Fig. 5). By further functional annotation and haplotype analysis (Supplementary Figs. 6–14), ten genes were prioritized as candidate genes responsible for ST-related traits, of which eight have been previously reported. For instance, *OsHKT1;5*^4^, *OsHKT2;3* (*OsHKT3*)^16^, *OsHAK2*^17,18^, and *OsHAK5*^18^ regulate Na^+^/K^+^ concentration ratio; *OsWRKY13*^19^ and *OsHAP2E*^20^ are involved in the regulation of osmotic homeostasis; *OsDSG1*^21^ and *OsBAHD*^22^ function on detoxification (Supplementary Figs. 3c, 6–12, Supplementary Tables 4 and 5, Supplementary Data 4–9, see Supplementary Note 3). We focused on characterizing the functions of *OsWRKY53* and *OsMKK10.2* associated with ST traits WC and SFW (Fig. 1G, H, Supplementary Figs. 3c, 15, Supplementary Note 4) and exploring their regulatory mechanisms on salt tolerance.

### *OsWRKY53* negatively regulates rice salt tolerance

**Fig. 2:**
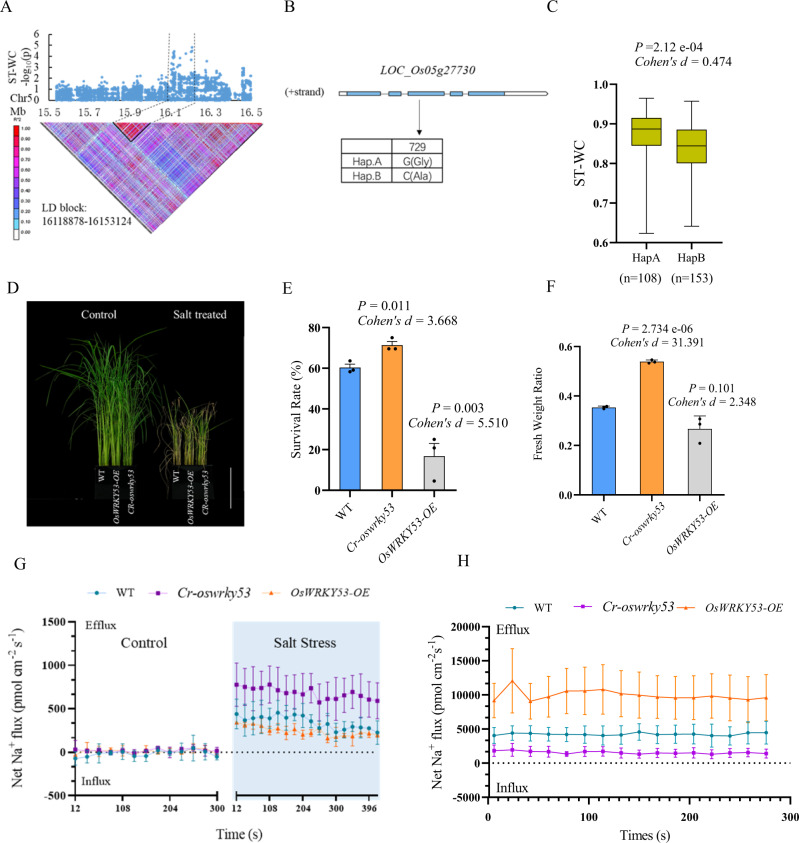
Identification and characterization of the causal gene *WRKY53*. **A** Local Manhattan plot (top) and LD heatmap (bottom) surrounding the peak on chromosome 5. **B** Exon-intron structure of OsWRKY53 and DNA polymorphisms in this gene. **C** Boxplot for ST-WC based on the OsWRKY53 haplotypes (Hap). Box edges represented the 0.25 and 0.75 quantiles with the medians shown by bold lines. Whiskers extended to data no more than 1.5 times the interquartile range. Differences between the haplotypes were analyzed by two-sided Student’s *t*-test. **D** Salt tolerance evaluation for WT (LG11), *OsWRKY53-OE*, and *Cr-oswrky53* mutant lines, without (left) and with 140 mM NaCl (right). Bar = 12 cm. **E** The fresh weight ratio (salt treatment/control) of WT, *OsWRKY53-OE*, and *Cr*-*oswrky53* under salt stress. Twelve seedlings were used to determine fresh weight and four seedlings were grouped randomly to calculate the fresh weight ratio shown as one data point. **F** Survival rates of WT, *OsWRKY53-OE*, and *Cr*-*oswrky53* under salt stress. Twenty-four plants were used to determine the survival rate. The values of survival rate and fresh weight ratio were significantly different from that of the WT. Data were presented as mean values ± SEM, *P* values were calculated with two-sided Student’s *t*-test, *n* = 3. **G** Net Na^+^ fluxes in 500 μm distance from root apex of WT, *OsWRKY53-OE*, and *Cr-oswrky53* measured using non-invasive micro-test technology under 140 mM NaCl treatment. **H** Net Na^+^ fluxes of the root xylem parenchyma cell in the roots of WT, *Cr-oswrky53*, and *OsWRKY53-OE* plants under the treatment of 140 mM NaCl for 24 h, *n* = 3, data were presented as means ± SEM. Source data were provided as a Source data file.

*OsWRKY53* located between 16.11 Mb-16.15 Mb on chromosome 5, and was associated with the water content (ST-WC) based on pairwise LD analysis (*r*^*2*^ > 0.8) (Fig. 2A). SNVs were found in the 5’UTR, coding region, and 3’UTR. Two haplotypes were identified based on the SNV in the coding sequence, and interestingly, rice varieties carrying haplotype A showed significantly higher WC (*P* = 2.12 e−04) than haplotype B under salt stress (Fig. 2B, C). Upon salt stress, the expression of *OsWRKY53* was transiently induced after 30 min (Supplementary Fig. 16a), indicating *OsWRKY53* might act as a salt-responsive signal. To validate the function of *OsWRKY53*, CRISPR knock-out line (*Cr-oswrky53*) and overexpression line (*OsWRKY53-OE*) were generated. Compared to WT, *Cr*-*oswrky53* displayed a higher fresh weight ratio and survival rate at l0 days post recovery from salt stress, while *OsWRKY53-OE* line showed a lower fresh weight ratio and survival rate (Fig. 2D–F). An independent CRISPR knock-out line of *Cr-oswrky53* also showed an increased survival rate under 140 mM NaCl treatment (Supplementary Fig. 16b–d). It suggested a negative role of OsWRKY53 in regulating ST-related traits. As ST trait WC was significantly correlated with shoot Na^+^ concentration (SNC) (*r* = −0.433**), we measured the Na^+^ effluxes in WT, *Cr-oswrky53*, and *OsWRKY53-OE*. Salt treatment caused significant fluctuation of Na^+^ flux in WT, *Cr-oswrky53*, and *OsWRKY53-OE* roots (Fig. 2G), but *Cr-oswrky53* roots showed higher Na^+^ efflux under salt stress during measuring period compared with WT. Likewise, *OsWRKY53-OE* roots exhibited lower Na^+^ efflux than WT and *Cr*-*oswrky53* roots. Furthermore, the Na^+^ fluxes of the parenchyma cell in WT, *Cr-oswrky53*, and *OsWRKY53-OE* were measured across the section of severed primary root. NMT analysis showed that Na^+^ flux of xylem parenchyma cell was significantly lower in *Cr-oswrky53* and higher in *OsWRKY53-OE* than that in WT (Fig. 2H, see Supplementary Note 5).

### *OsMKK10.2* functions as a positive regulator of salt tolerance

**Fig. 3:**
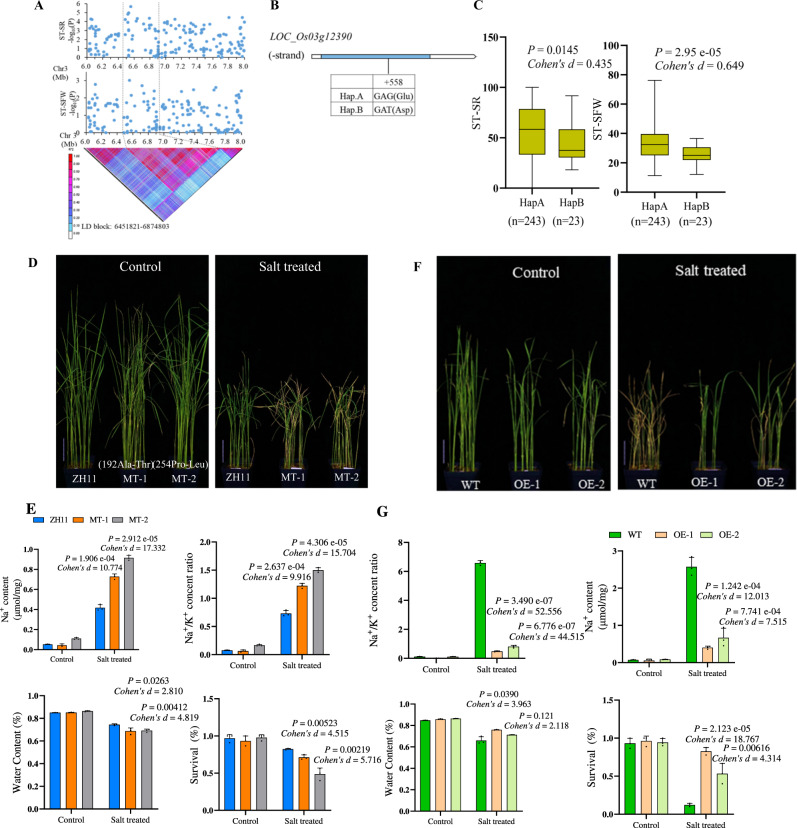
Identification and characterization of the causal gene *MKK10.2*. **A** Local Manhattan plot (top) and LD heatmap (bottom) surrounding the peak on chromosome 3. **B** Exon-intron structure of *MKK10.2* and DNA polymorphisms in this gene. **C** Boxplots for ST-SR based on the haplotypes (Hap) for *MKK10.2*. Box edges represented the 0.25 and 0.75 quantiles with the median values shown by bold lines. Whiskers extended to data no more than 1.5 times the interquartile range. Differences between the haplotypes were analyzed by two-sided Student’s *t*-test. **D** Evaluation of salt tolerance of WT ZH11, *mkk10.2* mutant plants. Pictures were taken 10 d post salt treatment, without (left) and with 200 mM NaCl (right). Scale bar, 5 cm. ZH11, Zhonghua11. **E** The shoot Na^+^ contents, shoot Na^+^/K^+^ content ratios, shoot water contents and survival rates in WT(ZH11), *mkk10.2* mutant plants under salt stress. The values were significantly different from that of the WT under 200 mM NaCl stress. Data were calculated 10 d post salt stress. Data were presented as mean values ± SD, *P* values were calculated with two-sided Student’s *t*-test, *n* = 3. **F** Evaluation of salt tolerance of WT, OE-1, and OE-2 (*OsMKK10.2OE-1* and *OsMKK10.2OE-2*) lines (bottom). Pictures were taken 10 d post salt treatment, without (left) and with 200 mM NaCl (right). Scale bar, 5 cm. **G** The shoot Na^+^ contents, shoot Na^+^/K^+^ content ratios, shoot water contents, and survival rates in WT, OE-1, and OE-2 (*OsMKK10.2OE-1* and *OsMKK10.2OE-2*) lines under salt stress. Data were calculated 10 d post salt stress without and with 200 mM NaCl. Significant differences were observed between WT and *OsMKK10.2*-OE lines under 200 mM NaCl stress. Data were presented as mean values ± SD, *P* values were calculated with two-sided Student’s *t*-test, *n* = 3. Twenty-four plants were used to determine the survival rate. Twelve seedlings were used to determine shoot Na^+^ content, shoot Na^+^/K^+^ content ratio and shoot water content, and each data point represented the mean value of four seedlings which were grouped randomly. Source data were provided as a Source data file.

*OsMKK10.2* locus was associated with ST trait survival rate (ST-SR) and predicted to reside on chromosome 3 at 6.5 Mb (Fig. 3A). Local linkage disequilibrium (LD) analysis showed a 420 kb LD block associated with salt tolerance. Although we identified 46 missense variants mapped to 20 genes through SNVs (Supplementary Data 9), haplotype analysis showed that only *OsMKK10.2* (*LOC_Os03g12390*), showed significant differences in ST-SR (Fig. 3C), whereas the remaining showed no significant differences (Supplementary Fig. 13). A SNV at base 6545857 of chromosome 3 caused a missense variation (Asp changed to Glu) in the coding sequence of *OsMKK10.2*. We next examined the kinase activities of OsMKK10.2^HapA^ and OsMKK10.2^HapB^, and found that the elite haplotype OsMKK10.2^HapA^ showed stronger kinase activity than OsMKK10.2^HapB^ (Supplementary Fig. 17a). The two haplotypes of this gene showed significant differences in ST-SR and shoot fresh weight (ST-SFW), whereas *OsMKK10.2*^*HapA*^ showed significantly higher ST-SR (*P* = 1.45 e−02) and ST-SFW (*P* = 2.95 e−05) under salt stress (Fig. 3B, C). *OsMKK10.2* expression was transiently induced by NaCl treatment after 4 h, and showed ~1.7-fold higher expression in the root of *OsMKK10.2*^*HapA*^ varieties as compared to that of *OsMKK10.2*^*HapB*^ varieties (Supplementary Fig. 17b), indicating that the expression of *OsMKK10.2* was positively associated with rice ST phenotypes. We next determined whether *OsMKK10.2* was required for salt tolerance by using two independent *OsMKK10.2* tilling mutants (*MT-1* and *MT-2*), a CRISPR knock-out line (*Cr-mkk10.2*) with eight-base deletion leading to a truncated kinase domain in *OsMKK10.2* (Supplementary Fig. 17c), and overexpression lines (*OsMKK10.2-OE-1* and *OsMKK10.2-OE-2*). Remarkably, the mutants *MT-1*, *MT-2*, and *Cr-mkk10.2* displayed salt-sensitive phenotypes of higher Na^+^ content and Na^+^/K^+^ content ratio under salt stress as compared to the parental line (Fig. 3D, E, Supplementary Fig. 17c–l). In contrast, *OsMKK10.2-OE* lines were more resistant to salt stress than WT as they exhibited significantly lower shoot Na^+^ content and shoot Na^+^/K^+^ content, but higher survival rate and shoot water content than WT under salt treatment (Fig. 3F, G). Furthermore, μ-XRF analysis showed that *Cr-mkk10.2* exhibited higher fluorescence intensity than wild type (Supplementary Fig. 18a, b). In the meantime, we examined the Na^+^ flux in *OsMKK10.2* knock-out line *Cr-mkk10.2* under salt stress. Our results revealed that the Na^+^ efflux was lower in *Cr-mkk10.2* compared to that in WT under NaCl treatment (Supplementary Fig. 18d). The Na^+^ fluxes in roots of WT (ZH11) and two tilling mutants of *OsMKK10.2* (*MT-1* and *MT-2*) were also measured. *MT-1* and *MT-2* mutants, resembling *Cr-mkk10.2*, showed lower Na^+^ effluxes than WT at 24 h post salt treatment (Supplementary Fig. 18e). These results demonstrated that *OsMKK10.2* played a positive role in salt tolerance through regulating Na^+^ efflux under salt stress.

### Functional interactions in the OsWRKY53-OsMKK10.2 cascade

Since *OsWRKY53* and *OsMKK10.2* both regulated Na^+^ efflux, we questioned whether OsWRKY53 and OsMKK10.2 regulated the ion transporters such as SOS1 and HKT1;5 which was also identified to mediate ST traits by our GWAS analysis. To address it, we examined all interaction combinations among the ST-related genes.

By performing yeast one-hybrid assays, we found that *OsWRKY53* could bind the promoter of *OsMKK10.2* (Supplementary Fig. 19a). The bind of OsWRKY53 to the promoter region of *OsMKK10.2* was further confirmed by Chromatin immunoprecipitation (ChIP) assay (Supplementary Fig. 19b). Subsequent Surface Plasmon Resonance (SPR) experiment (Supplementary Fig. 19c) and Electrophoretic Mobility Shift Assay (EMSA, Supplementary Fig. 19d) showed that specifically bound to the W-box element of the *OsMKK10.2* promoter, and the binding activity of OsWRKY53 to the promoter of the *OsMKK10.2* depended on the protein concentration (5.735–86.00 nM). Moreover, the dissociation rate constant KD of OsWRKY53 to the promoter of the *OsMKK10.2* was observed at 9.586 E-9 M, strongly suggesting that immobilized OsWRKY53 could tightly bind the promoter of *OsMKK10.2*. Altogether, these biochemistry experiments demonstrated a tight molecular interaction between *OsWRKY53* and *OsMKK10.2* in salt tolerance.

OsWRKY53 can bind to the W-box motif^23^, and the promoter of the *OsMKK10.2* contain one W-box element. There was no difference in binding site detected in the promoters of *OsMKK10.2* haplotypes, while amino-acid sequence varied between the *OsMKK10.2* haplotypes due to a single SNV (G changed to T) (Fig. 3B).

We next tested the capacity of OsWRKY53 protein on driving the expression of *OsMKK10.2* by conducting dual-luciferase assays in *planta*. The promoter of *OsMKK10.2* was fused to the luciferase reporter gene and co-transformed into rice protoplast with constructs driving the expression of OsWRKY53 (Supplementary Fig. 19e, f). LUC activity driven by the promoter of *OsMKK10.2* was severely decreased in the presence of OsWRKY53 as compared to the control, and the binding activity of OsWRKY53 to the promoter of *OsMKK10.2* was attenuated without the W-box element (Supplementary Fig. 19g), implying that OsWRKY53 trans-repressed the expression of *OsMKK10.2*.

### OsWRKY53 trans-represses *OsHKT1;5* under salt stress

**Fig. 4:**
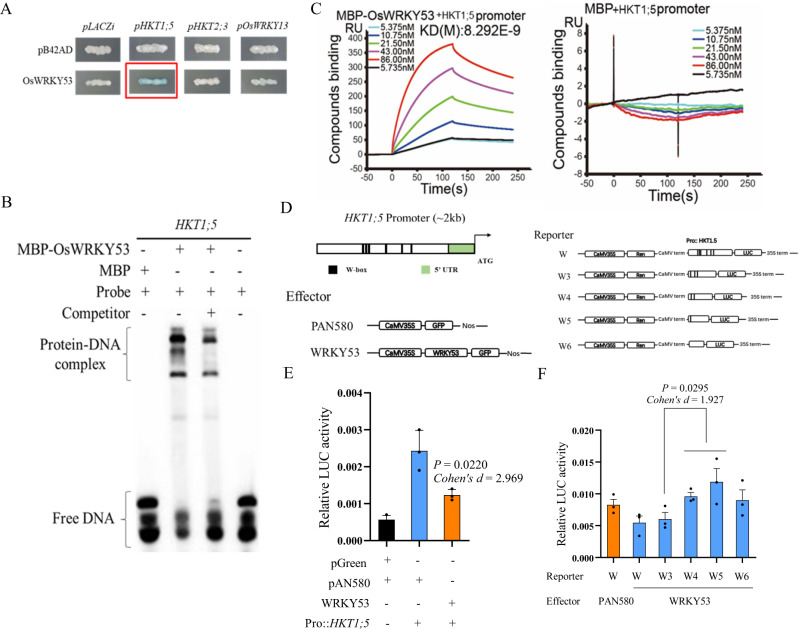
Interactions between *OsWRKY53* and *OsHKT1;5* in salt tolerance. **A** Yeast one-hybrid assays to study interaction between transcription factor OsWRKY53 and target gene *OsHKT1;5*. **B** DNA binding activity of OsWRKY53 protein on *OsHKT1;5* promoter fragment tested by EMSA assays. Brackets showed protein-DNA complex or free probe, respectively. EMSA assays were repeated three times. MBP-OsWRKY53 fusion protein was expressed in *E. coli*. The probes were labeled with biotin. **C** OsWRKY53 binding to W-box element of *OsHKT1;5* promoter in surface plasmon resonance (SPR) experiments by using Biacore T200 instrument. **D** Different binding sites of transcription factor in the promoter of *OsHKT1;5* and schematic representation of constructs used in transcriptional activity assay. **E** OsWRKY53 trans-repressed *OsHKT1;5*. Dual-luciferase reporter assay was used to measure luciferase activity. **F** Truncated promoters harboring different number of W-box were generated to drive firefly luciferase gene. Renilla luciferase gene was used as an internal control. Data were normalized to the internal control 35 S::REN. The values were significantly different from that of the control. Data were presented as means ± SD. *P* values were calculated with two-sided Student’s *t*-test, *n* = 3. Source data were provided as a Source data file.

The above yeast one-hybrid assay also demonstrated that *OsWRKY53* could bind the promoter of *OsHKT1;5* (Fig. 4A). *OsHKT1;5* encoded a Na^+^ transporter of HKT family class I and was reported to promote rice salt tolerance through mediating Na^+^ transport in xylem and phloem parenchyma cell^4^. The bind of OsWRKY53 to the promoter of *OsHKT1;5* was further confirmed by ChIP assay (Supplementary Fig. 20a). A set of biochemistry experiments, including EMSA and SPR experiments, showed that *OsWRKY53* specifically bound to the W-box elements of the *OsHKT1;5* promoter (Fig. 4B, C). The binding activity also depended on the protein concentration (5.735–86.00 nM), with a dissociation rate constant KD of OsWRKY53 to the *OsHKT1;5* promoter at 8.292 E-9 M, strongly confirming that immobilized OsWRKY53 tightly bound the promoter of the *OsHKT1;5* gene (Fig. 4C). Hence, these biochemistry experiments demonstrated the tight molecular interactions between *OsWRKY53* and *OsHKT1;5* in salt tolerance.

We next tested the capacity of OsWRKY53 protein on driving the expression of *OsHKT1;5* by conducting dual-luciferase assays *in planta*. The promoter of *OsHKT1;5* was fused to the luciferase reporter gene and co-introduced into rice protoplast with constructs driving the expression of OsWRKY53 (Fig. 4E, and Supplementary Fig. 20b). In the presence of OsWRKY53, LUC activity driven by the promoter of *OsHKT1;5* was largely reduced as compared to control, suggesting that OsWRKY53 trans-repressed the expression of *OsHKT1;5* (Fig. 4E). We generated four truncated *OsHKT1;5* promoter-reporter constructs (W3, W4, W5, and W6) and the construct containing all the six W-box elements (W) was used as a control. We found that deletion of first three w-boxes did not affect the luciferase activity. When the fourth W-box was removed, WRKY53-mediated transcriptional repression was relieved (Fig. 4F). These results indicated that the fourth W-box element in *OsHKT1;5* promoter contributed to the binding activity of OsWRKY53 to the promoter of *OsHKT1;5*.

Consistent with physical and genetic interactions between OsWRKY53 and OsHKT1;5, we also observed that the salt-induced *OsHKT1;5* expression in root tissue was enhanced in the *Cr-oswrky53* mutant (Supplementary Fig. 20c), confirming a regulation role of *OsWRKY53* in *OsHKT1;5* expression under salt stress. NMT analysis further showed that Na^+^ flux of xylem parenchyma cell was significantly lower in *Cr-oswrky53*, and higher in *OsWRKY53-OE*, as compared to that in WT (Fig. 2H). OsHKT1;5 transported Na^+^ from the xylem stream to parenchyma cell in the root elongation zone as previously reported^4^. Therefore, low Na^+^ efflux in the parenchyma cell of *Cr-oswrky53* was due to strong Na^+^ transport by OsHKT1;5, indicating that OsHKT1;5 activity was higher in *Cr-oswrky53* than WT and *OsWRKY53-OE*. Our results suggested that *OsWRKY53* mediated salt tolerance through the transcriptional repression of ST gene *OsHKT1;5*.

### OsWRKY53-OsHKT1;5 and OsWRKY53-OsMKK10.2 confer salt-tolerance

We constructed CSSLs (Chromosome segment substitution lines) using Youzhan 8 as the recipient parent and *O*.*rufipogon Griff*. as donor parent. We compared the salt tolerances of CSSL (*OsWRKY53-OsMKK10.2*) and CSSL (*OsWRKY53-OsHKT1;5*). As a result, CSSL(*OsWRKY53-OsMKK10.2*) was more sensitive to salt stress compared to CSSL (*OsWRKY53-OsHKT1;5*) under NaCl treatment (Supplementary Fig. 21a). Therefore, *OsWRKY53-OsMKK10.2* cascade could be the major contributor to salt tolerance. We further genetically knocked-out either *OsMKK10.2* or *OsHKT1;5* in the background of *Cr-oswrky53*, and evaluated the survival rates under salt stress. We found that *Cr-wrky53hkt1;5* double knock-out mutant partially rescued the phenotype of *Cr-oswrky53*, while *Cr-wrky53mkk10.2* completely rescued the phenotype of *Cr-oswrky53* and exhibited sever salt sensitivity as compared to *Cr-oswrky53* and WT (Supplementary Fig. 21e). These evidences further supported that *OsMKK10.2* and *OsHKT1;5* acted downstream of *OsWRKY53* to regulate salt tolerance, and OsWRKY53-OsMKK10.2 cascade contribute more to salt tolerance due to the biological significance of OsMKK10.2 in salt tolerance.

It has been reported that OsWRKY53 acts as a negative modulator of MPK activity, and suppresses herbivore-induced pest defenses^24^. OsMKK10.2 can promote disease resistance by activating multiple MPKs including OsMPK6 in rice^25^. AtMPK6 can phosphorylate and activate the SOS1 Na^+^/H^+^ antiporter activity^15^. In epidermis surrounding the parenchyma cells of roots, SOS1 encodes a plasma membrane Na^+^/H^+^ antiporter, which uses the H^+^ gradient to drive Na^+^ efflux and thus reducing cytosolic Na^+^ concentration^26^. We have found that OsWRKY53-OsMKK10.2 modulated ion homeostasis, so we speculated that the OsWRKY53-OsMKK10.2 module might activate OsMPK6 in salt stress.

### OsMKK10.2 activates OsMPK6 to regulate salt tolerance

**Fig. 5:**
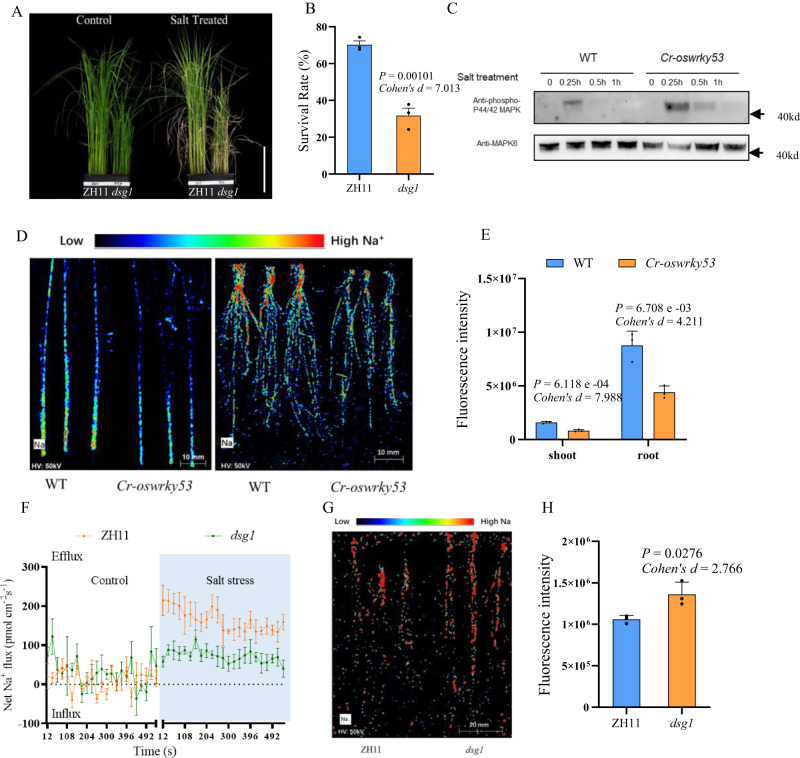
The mutant of *mpk6* (*dsg1*) displayed salt-sensitive phenotypes. **A** Phenotypes of wild type (ZH11) and MPK6 mutant *dsg1* under 0 mM and 140 mM NaCl. Bar = 12 cm. **B** Survival rate of *dsg1* under 140 mM NaCl for 2 weeks. Thirty-two plants were used to determine the survival rate. **C** Phosphorylation level of MPK6 in *Cr-oswrky53* compared with wild type during salt treatment. MPK6 was used as a control. Phosphorylation detection in vivo was repeated three times. **D** The images of μ-XRF of root and shoot in WT and *Cr*-*oswrky53* under 140 mM NaCl, respectively. **E** Fluorescence intensity of μ-XRF scan in root and shoot of WT, *Cr*-*oswrky53* under 140 mM NaCl shown in (**D**), *n* = 3. Data were presented as means ± SD. *P* values were calculated with two-sided Student’s *t*-test. **F** Mean Na^+^ fluxes in 500 μm distance from root apex of WT and *dsg1* under 140 mM NaCl. **G** The image of μ-XRF of root in WT and *dsg1* under 140 mM NaCl, respectively. **H** Fluorescence intensity of μ-XRF scan in root of WT and *dsg1* under 140 mM NaCl shown in (**G**). Data were presented as means ± SD. *P* values were calculated with two-sided Student’s *t*-test, *n* = 3. Source data were provided as a Source data file.

To verify this possibility, we obtained the mutant of *OsMPK6* (*dsg1*) with a weak *mpk6* allele^27^. Compared with WT, *dsg1* exhibited salt-sensitive phenotype under NaCl treatment with a lower survival rate (Fig. 5A, B). The expression of *OsMPK6* was also induced instantly by NaCl treatment, similar to the expression of *OsMKK10.2* (Supplementary Fig. 22a, b), while protein level of OsMPK6 in *dsg1* decreased significantly under NaCl treatment for 5 h (Supplementary Fig. 22c).

We expressed wild-type OsMKK10.2, mutant OsMKK10.2 (*Cr-mkk10.2*) and OsMPK6 in *E. coli*. In vitro kinase assay was operated to confirm that *Cr-mkk10.2* failed to phosphorylate MPK6 (Supplementary Fig. 23a). We next examined the phosphorylation levels of OsMPK6 in *Cr-oswrky53* and *Cr-mkk10.2* under NaCl treatment. Phosphorylated OsMPK6 was increased immediately under salt treatment (15 min and 30 min) in WT seedlings, which was significantly impaired in *Cr-mkk10.2* (Supplementary Fig. 23b). While in *Cr-oswrky53*, phosphorylated OsMPK6 was significantly increased at 15 min and 30 min post salt treatment compared with WT (Fig. 5C). These results suggested that OsWRKY53 negatively regulated the phosphorylation level of OsMPK6.

The Na^+^ contents in WT and *Cr-oswrky53* were further determined by μ-XRF. Compared with WT, *Cr-oswrky53* showed lower Na fluorescence intensity in both root and shoot (Fig. 5D, E). These data indicated that OsWRKY53 negatively regulated salt tolerance through affecting Na^+^ efflux. Furthermore, measurement of Na^+^ flux showed that Na^+^ efflux was significantly decreased in *dsg1* compared with WT (Fig. 5F). In addition, μ-XRF analysis also showed a higher Na fluorescence intensity in *dsg1* than WT (Fig. 5G, H).

Taken together, our results suggested that OsWRKY53-OsMKK10.2 cascade negatively regulated the phosphorylation of OsMPK6-OsSOS1 to mediate salt tolerance in rice.

### Selective sweep and domestication of *OsSKC1*^*HapA*^

**Fig. 6:**
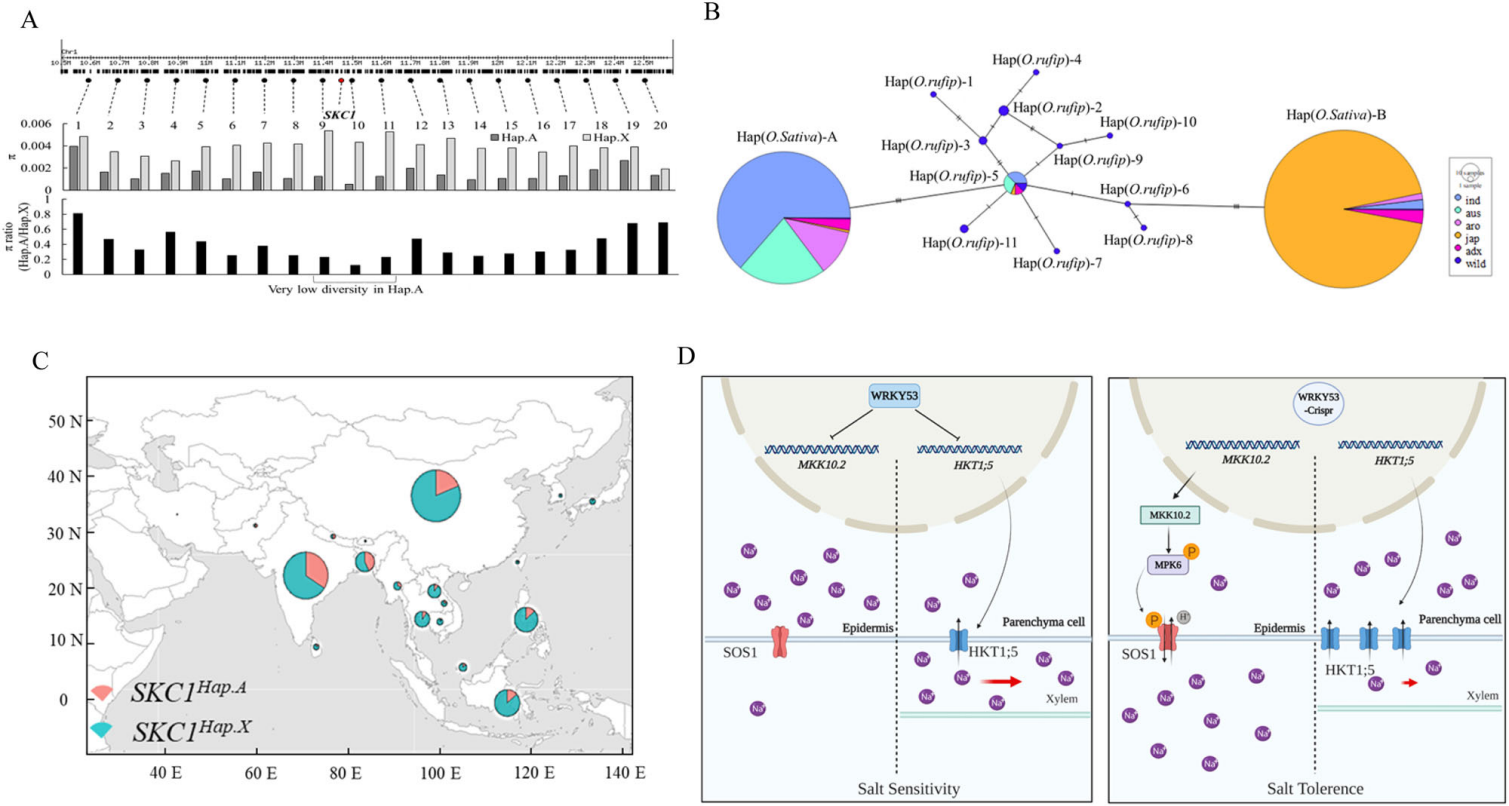
The salt tolerance rice-SKC1^HapA^ was derived from variation in *O. rufipogon* and selected in *O. sativa*. **A** Nucleotide diversity across the SKC1(HKT1;5) genomic region. Top: the 21 sampled loci (including SKC1) located in the genomic region around the SKC1 gene on chromosome 1. Middle: nucleotide diversity pi of Hap X and A rice at the sampled loci. Bottom: the relative ratio of pi in HapA rice to HapX rice showed a selective sweep of ~300 kb surrounding SKC1 in HapA rice. **B** Haplotype network of the SKC1 gene. Each haplotype was separated by mutational changes, with hatches indicating mutational differences between linked haplotypes. aro, *aromatic*; tej, *temperate japonica*; trj, *tropical japonica*; adx, *admix*; wild, *O. rufipogon*. **C** Geographical distribution of the SKC1^HapA^ in rice 3 K. The pie chart size was proportional to the number of accessions. **D** A model for OsWRKY53 trans-regulating OsMKK10.2-OsMPK6-OsSOS1 cascade and OsHKT1;5 in response to salt stress. OsWRKY53 trans-repressed *OsMKK10.2* and *OsHKT1;5* showing salt sensitivity (left), while WRKY53-CRISPR enhanced OsMKK10.2-OsMPK6-OsSOS1 and OsHKT1;5 conferring salt tolerance (right).

Our GWAS analysis showed that *OsHKT1;5* (*SKC1*) was associated with shoot K^+^ concentration (SKC). *SKC1* could be classified to two haplotypes by variants in its coding region, and *SKC1*^*HapA*^ showed stronger salt tolerance than *SKC1*^*HapB*^ in SKC and shoot height (SH) under salt treatment (Supplementary Fig. 6). We calculated the nucleotide diversity within a ~2 Mb interval surrounding the gene and observed an interval of ~300 kb surrounding *SKC1* with significantly reduced nucleotide diversity in *SKC1*^*HapA*^ varieties relative to *SKC1*^*HapX*^ varieties (varieties do not harboring *SKC1*^*HapA*^ in rice3K population) using rice3K data (Fig. 6A). Nucleotide diversity was not reduced in the flanking regions of *OsWRKY53* or *OsMKK10.2*, showing no selective sweep signal. Therefore, we further investigated *SKC1*^*HapA*^ presence in *O. sativa*’s wild ancestor, *Oryza rufipogon*, to study the domestication of *SKC1*. Gene haplotype network analysis showed that wild and cultivated rice shared *SKC1*^*HapA*^. *SKC1*^*HapA*^ emerged in indica rice during Hap (*O. rufipogon*)-5 differentiation, and *SKC1*^*HapB*^ emerged in japonica rice during Hap (*O. rufipogon*)-6 differentiation (Fig. 6B). Most of Hap (*O. rufipogon*)-5 originated from Bangladesh and Jiangxi province of China. In the Rice3K database (http://snp-seek.irri.org/), there were 582 varieties harboring *SKC1*^*HapA*^. Occurrence was more frequent in India, Bangladesh, and China. The salt tolerance rice-*SKC1*^*HapA*^ was selected for salinity tolerance rice cultivation in these regions (Fig. 6C). Our results suggested that the salt tolerance rice-*SKC1*^*HapA*^ was derived from variation in *O. rufipogon* and selected in *O. sativa* for conferring salinity tolerance in large area of salt soil.

We proposed a possible working model for the network (Fig. 6D), in which *OsWRKY53*, as a negative modulator of salt tolerance, plays an essential role under salt stress through regulating ion homeostasis.

## Discussion

In the study, we identify a transcriptional factor OsWRKY53 and its controlled signaling cascades conferring salt tolerance in rice. We demonstrate that OsWRKY53 could directly regulate the expressions of *OsMKK10.2* and *OsHKT1;5* to regulate ST-related traits, highlighting a central role of OsWRKY*53* in salt tolerance. These results are critical to enrich the mechanism underlying salt-tolerance.

Our OsWRKY53-regulated network establishes a connection between upstream regulator and OsMPK cascades that was critical to the protection of rice from salinity stress. OsWRKY53-regulated OsMKK10.2 that participate in salt tolerance. Moreover, OsWRKY53 can be phosphorylated by OsMPK6 to regulate rice seed size^28^. We found that loss of OsWRKY53 function activated OsMKK10.2 and OsMPK6 constantly and broke the balance between growth and stress-resistance because *Cr-oswrky53* exhibited weaker growth characters than WT at both the seedling and mature stages (Supplementary Fig. 24). We therefore speculate that OsMPK6 participates in salt response (Supplementary Fig. 23b) and activates OsWRKY53 to weaken OsMKK10.2-OsMPK6 signaling and protect plant from overreacting to salinity.

As contradiction grows between the increasing of population and limited arable land for agriculture, the discovery of genetic sources for stress tolerant rice might provide adaptive solution to modern rice breeding. The WRKY53-regulated network, modulating both SOS1 Na^+^/H^+^ antiporter and HKT1;5 Na^+^ transporter, provides the endogenous cues for rice to adapt to environment stimulus, and further paves the way for future investigation of the detailed biochemical mechanisms protecting rice plants and also other plant species from broad stresses from salinity. In summary, the discovery of the gene network modulated by *OsWRKY53* provides an avenue for protecting rice plants from salinity for a more sustainable agriculture.

## Methods

### Phenotyping and genotyping-by-sequencing

A total of 268 rice accessions were used as the plant materials. The seeds of all accessions were collected, stored, and supplied by State Key Laboratory of Crop Genetics and Germplasm Enhancement, Nanjing Agricultural University, China (Supplementary Data 2). Rice seeds were rinsed with sterile water and placed on filter paper soaked in water and allowed to germinate for 3 days at 28 °C. Germinated seedlings were transferred to a hydroponic system. Plants were grown in a growth-chamber at 28 °C/26 °C in a 12 h photoperiod regime and with 60% humidity. The greenhouse experiment adopted a completely random block design with three replicates. The salt treatment group and the control group were set up in two groups by random block design. The salt stress was imposed on the seedlings with two and one-half leaves by supplementing with 0 mM as control and 140 mM NaCl as salt treatment. The solutions were renewed every 2 days and the pH was maintained at 5–6. The shoots of seedlings were sampled, and their shoot height (SH), shoot fresh weight (SFW), shoot dry weight (SDW), water content (WC), shoot Na^+^ concentration (SNC), shoot K^+^ concentration (SKC) and shoot Na^+^/K^+^ concentration ratios (SNKR) were measured after 10 days of treatment. After that, all plants were transferred to fresh Yoshida’s solution to recover for 10 days and then the survival rates (SR) were determined. In order to obtain accurate phenotypic data, three biological replicates were set up in the treatment group and the control group, and four plants were selected from each replicate for measurement. The criteria for selection were the four plants located in the block.

Genomic DNA was extracted from the 268 rice accessions. DNA was extracted from leaf tissues using the DNeasy Plant Mini Kit (Qiagen, Germany). GBS library of the 268 varieties was prepared for single end-sequencing according to Tang et al.^29^. Briefly, a total of 2 μg genomic DNA from each accession was digested for 1 h at 37 °C in a 50-μL reaction with 50 U of EcoRI (New England Biolabs). Barcodes were 6-bp long, being at least two mutational steps separated from each other. The GBS library was constructed and sequenced on an Illumina Hiseq2500. Reads were separated by barcode and trimmed at the 3’ ends. The raw reads of high quality were assembled based on the genomic sequences of the japonica rice cultivars Nipponbare using TMAP3.6. SNVs of each sample were collected using the TASSEL pipeline^30^. A total of 81,615 polymorphic SNVs were identified in the sequence data that segregated in the population and 40,889 passed quality control process. The SNV cladogram-tree dataset was generated using the neighbor-joining method in TASSEL^30^. Distinct groups were identified by principal component analysis using TASSEL^31^.

### Construction of reference panel and genotype imputation

We first downloaded Rice3K reference genomes in plink format with 3024 samples and a total of 29,635,224 biallelic SNVs from public database (https://snpseek.irri.org/_download.zul). We then conducted quality control on the data by including SNVs with MAF >= 0.05 and with a 95% genotyping rate (5% missing). The missing genotypes in the reference data were imputed by fastPhase so that fully genotypic reference dataset was constructed. SHAPEIT2 was then used to perform the pre-phasing step with an accurate phasing method.

IMPUTE2 (version 2.3.2) was used for imputation with default parameters. We performed imputation by splitting chromosome in chunks of equal size 5 MB as recommended. To avoid margin effects while chunking genotypic region, IMPUTE2 used an internal buffer region (default is 250 kb) on either side of the analysis interval. Imputation processes were run in a parallel way to speed up the computational runtime. At the end of each computation, we extracted the imputation quality scores (info). We removed any SNVs whose info metric <0.7 in order to obtain imputed SNVs with high certainty.

### Population genetics and genome-wide association analyses

The number of subpopulations (K) was determined using STRUCTURE version 2.3.4^32^. We ran 10,000 iterations, and the number of clusters (K) was set from 2 to 10. Each accession was assigned to a subpopulation for which the membership value (Q value) was the maximum value^33^. Principal component analysis (PCA) of whole-genome imputed SNVs was performed with the TASSEL software, and the first three eigenvectors were plotted in three dimensions. The SNV cladogram-tree dataset was generated using the neighbor-joining method as provided in TASSEL 5.2.13.

A mixed linear model program in TASSEL was used for the association analysis. In order to eliminate false positives in association analysis, population structure was used as a fixed effect, while relatedness was used as a random effect. Population structure matrix Q was calculated using admixture, which was used as fixed effect in the mixed model to correct for stratification. The random effect was estimated from the groups clustered based on the kinship among all accessions.

### Strategy of prioritizing candidate genes

Firstly, we regarded the imputed SNVs which were significantly associated with target traits of interest at suggestive threshold *P* value < 10^−4^ as promising variants. Secondly, we remove loci whose LD block has no gene through linkage disequilibrium analysis and functional annotation. Thirdly, further filter through searching related rice database, pathways, and literature. Lastly, perform real-time PCR (RT-PCR) for multiple potential candidate genes within a LD block then obtain candidate causal gene for follow-up functional validation through complementation tests.

### Real-time PCR

To investigate expressions of the salt tolerance genes, total RNA was isolated using plant RNA purification reagent (Invitrogen). Real-time PCR was done in Real-Time PCR machine (I-Cycle, Bio-Rad), with each reaction containing 200 ng of first-strand cDNA, 0.5 μL of 10 mmol L^−1^ gene-specific primers, and 12.5 μL of real-time PCR SYBR MIX (iQ™ SYBR® Green Supermix, Bio-Rad). Amplification conditions were 95 °C for 5 min followed by 40 cycles of 95 °C for 15 s and 60 °C for 60 s. The rice Actin was selected as the endogenous reference. The PCR specificity was examined by 3% agarose gel using 5 µL from each reaction to check the right product length and make sure no primer dimer or non-specific amplicons. The primers for real-time PCR together with cDNA amplification were listed in Supplementary Data 10.

### Functional validation and complemental experiment

The *mkk10.2* mutant was obtained from the Crop Tilling Mutant Database of Prof. Chunming Liu, Key Laboratory of Plant Molecular Physiology, CAS (http://www.croptilling.org)^34^. The WT was ZH11, a japonica cultivar. To identify the mutant locus in mkk10.2, we tested mutations through the PCR amplification and sequencing. To obtain the OE transformation lines of *MKK10.2*, full-length cDNA of *MKK10.2* was amplified by PCR and cloned into the vector pCUbi1390 with the maize Ubi promoter, which was then transformed into the rice variety Ningjing4 by the Agrobacterium-mediated method.

### Determination of Na^+^ and K^+^ Content

Na^+^ and K^+^ concentration were determined according to the method of Wang et al.^35^ with minor modifications. In brief, the shoots of seedlings were sampled after 10 d of salt treatment. The samples were dried at 70 °C for 2 d, and 100 mg of dried tissue was digested with 5 mL of nitric acid at 50 °C for 30 min and 90 °C for 4 h for digested completely, diluted to 25 mL with distilled water after cooling it down. Take 2 mL of the liquid samples and dilute it to 10 mL. Na^+^ and K^+^ ion contents analysis were carried out with ICP-OES (Optima 8000; PerkinElmer).

### Yeast one-hybrid assay

All procedures were performed according to the manufacturer’s protocol (Clontech). *OsWRKY53* and *OsWRKY13* full-length cDNA were cloned into the pB42AD vector, which includes a B42 transcriptional activation domain. Approximately 2 kb of the *MKK10.2*, *HKT1;5*, *OsHKT2;3,* and *WRKY13* promoters were cloned separately into the lacZ (β-galactosidase) reporter plasmid pLACZi. All clones were sequenced to ensure that the sequences were accurate. Yeast strain EGY48 (MATα, trp1, his3, ura3, leu2::6 LexAopsLEU2; Invitrogen) was used for transformation. Yeast strain EGY48 was transformed with one of the 2 proteins vector and one of the 4 reporter plasmids per transformation. Interactions were tested on selection plates (SD/–Ura–Trp+X-gal).

### Electrophoretic mobility shift assay (EMSA)

The MBP-OsWRKY53 fusion protein was expressed in *E. coli* pMal-c2x at 16 °C for 20–24 h in the presence of 0.1 mM isopropyl-b-D-1-thiogalactopyranoside. MBP-OsWRKY53 protein was purified using Amylose Resin (New England Biolabs) according to the manufacturer’s instructions. *MKK10.2* and *HKT1;5* promoter fragments each containing 48 bp were synthesized using EMSA Prob Biotin Labeling Kit. The LightShift™ Chemiluminescent EMSA Kit was used to perform EMSA following the manufacturer’s instructions. Sixty fmol of biotin-labeled DNA probes was incubated with 2 μg of purified proteins (MBP-OsWRKY53) in a total volume of 20 μL. The reaction mixtures were incubated at 25 °C for 30 min and loaded onto a 6% (w/v) native polyacrylamide gel. Electrophoresis was conducted at 100 V for 1.5 h in 0.5×TBE buffer (44.5 mM Tris, 44.5 mM boric acid, and 1 mM EDTA, pH 8.3) at 4 °C. The gel was sandwiched and transferred to a positively charged Nylon Membranes (Roche) in 0.25×TBE buffer at 200 mA for 45 min at 4 °C. Biotin-labeled DNA was detected using Chemiluminescent Nucleic Acid Detection Module Kit (Thermo Scientific) and X-ray film.

### Surface plasmon resonance analysis

Biacore T200 and CM5 chips (GE Healthcare) with cross-linked anti-biotin rabbit polyclonal antibody (Abcam, ab1227, 1:10,000 dilution) were used for all SPR experiments. The instrument was first primed three times with reaction buffer and flow cell 1 (FC1) was used as the reference flow cell, which was unmodified and lacked the oligonucleotide. Flow cell 2 (FC2) was used for the immobilization of the oligonucleotide. The biotin-labeled oligonucleotide was injected over a 1 min period at a flow rate of 5 μL min^−1^, and immobilization levels of 200–300 RU were routinely observed under these conditions. Protein-DNA binding assays were performed in the reaction buffer at the relatively high flow rate of 10 μL min^−1^ to avoid or minimize any mass-transport limitation effects. Protein solutions (15, 30, 60, 120, and 240 nM) were injected for 120 s followed by a dissociation in reaction buffer for 60 s. At the end of the dissociation period, the sensor chip was regenerated to remove any remaining bound material by injecting reaction buffer, containing 15 mM NaOH, at 30 μL min^−1^ for 300 s.

### ChIP assay

OsWRKY53-OE was used for ChIP assay with the Millipore ChIP Assay Kit (17-295; Millipore). Briefly, ~4 g of rice seedlings was cross-linked in 1% formaldehyde under a vacuum for 10 min, and cross-linking was stopped with 0.1 M Gly. The sample was ground to a powder in liquid nitrogen and used to isolate nuclei. Chromatin was sonicated to an average fragment size of 200–500 bp. Anti-OsWRKY53 (3:200 dilutions) was used to immunoprecipitate the protein-DNA complex, and the precipitated DNA was recovered and analyzed by quantitative PCR. Chromatin precipitated without antibody was used as a control^36^. AntiOsWRKY53 (AbP80095-A-SE) was ordered from Beijing Protein innovation. Primers used for ChIP-qPCR are listed in Supplementary Data 10.

### Dual-luciferase assay

Full-length ORF of *OsWRKY53* was cloned into pAN580GFP to act as the effector. The promoter fragments of *OsMKK10.2*, *OsSKC1* were fused into pGreenII0800-LUC to obtain two reporters. Transient co-expression of the effector and reporter constructs were transfected into rice protoplast for 20 h. Briefly, LUC and REN activities were surveyed with a Dual-Luciferase reporter assay kit (Promega), and the LUC activity, normalized to REN activity, was determined. The *Renilla* luciferase (REN) gene driven by 35 S promoter was used as an internal control. Transient transactivation with the reporters and the empty vector pAN580GFP was used as controls. All primers used for these constructs are listed in Supplementary Data 10.

### In vitro kinase assay

The full-length of GST-MKK10.2 and MBP-MPK6 fusion proteins were expressed and purified via *E. coli* strain BL21 (DE3) by standard protocols using amylose agarose beads and glutathione magnetic beads (Solarbio), respectively. For in vitro kinase assay, fusion proteins were incubated with 25 mM Tris-HCl (pH 7.4), 12 mM MgCl_2_, 1 mM DTT, and 1 mM ATP at 30 °C for 45 min and stopped by SDS loading buffer. MPK6 phosphorylation level was evaluated using anti-phospho-p44/42 MAPK polyclonal antibody (Cell Signaling Technology, #9101, 1:2000 dilution). Loading controls of GST-MKK10.2, MBP-MPK6 were detected using anti-GST (Beijing Protein Innovation, AbM59001-2H5-PU, 1:5000 dilution), anti-MAPK6 (Beijing Protein Innovation, AbP80140-A-SE, 1:2000 dilution), respectively.

### Selective sweep analysis

A haplotype network construction of *OsHKT1;5* was generated using PopART version 1.7^37^. DnaSP version 4.0^38^ was used to calculate total nucleotide diversity per nucleotide site (π), relative ratio of π, and selective sweep signals^39,40^. The geographical distribution of the accessions with *OsHKT1;5*^*HapA*^ was drawn using R software.

### Root Na^+^ flux analysis

The Net Na^+^ flux was measured using NMT (Non-invasive Micro-test Technology, Xuyue Sci. & Tech. Co. Ltd., Beijing, China). Seedlings were fixed under Na^+^ ion-selective microelectrode in measuring solution (0.1 mM KCl, 0.1 mM CaCl_2_, 0.1 mM MgCl_2_, 0.5 mM NaCl, 0.3 mM MES, 0.2 mM Na_2_SO_4_, pH = 6). Net Na^+^ efflux scan (Supplementary Fig. 18c) in different distance from root apex was used to confirm the maximum Na^+^ flux region (500 μm distance from root apex). Na^+^ ion-selective microelectrodes were propelled to ~5 μM from root elongation zone. Na^+^ flux was measured for more than 5 mins.

### Micro X-ray fluorescence (μ-XRF) analysis

The μ-XRF was performed on a μXRF spectrometer (M4 Tornado plus, BRUKER), operated at a voltage of 50 kV and an anode current of 600 µA. Each mapping was measured with a spot distance of 9 µm and a spot measuring time of 2 ms. Seven days rice seedlings treated under 140 mM NaCl for 2 days were used for μ-XRF analysis and vacuum was applied to the sample chamber before μXRF analysis was performed.

### Reporting summary

Further information on research design is available in the Nature Portfolio Reporting Summary linked to this article.

## Supplementary information

Supplementary Information

Description of Additional Supplementary Files

Supplementary Data 1

Supplementary Data 2

Supplementary Data 3

Supplementary Data 4

Supplementary Data 5

Supplementary Data 6

Supplementary Data 7

Supplementary Data 8

Supplementary Data 9

Supplementary Data 10

Reporting Summary

## Data Availability

Data supporting the findings of this work are available within the paper and its Supplementary Information files. A reporting summary for this Article is available as a Supplementary Information file. The GBS data have uploaded to NCBI SRA (Sequence Read Archive) database (SRR13518263, SRR13493593, SRR13493703, and SRR13495248). Rice3K genomic SNPs can be downloaded from the Rice SNP-Seek Database [https://snp-seek.irri.org/_download.zul;jsessionid=1473933665C82060834E97789296DB2A]. The data of the 42 wild rice genomic SNPs used in the haplotype network analysis can be downloaded from NCBI [https://www.ncbi.nlm.nih.gov/bioproject/PRJNA407820]. Source data are provided with this paper.

## Source data

Source Data
