# Supplementary material for: Genome-wide association studies identify OsWRKY53 as a key regulator of salt tolerance in rice
Source: Nat Commun. 2023 Jun 15;14:3550. doi: 10.1038/s41467-023-39167-0 (PMC10272163; doi:10.1038/s41467-023-39167-0)
Supplement: Supplementary file 1 — Supplementary Information [file 41467_2023_39167_MOESM1_ESM.pdf]

Genome-wide association studies identify OsWRKY53 as a key regulator of salt tolerance in rice

Yu *et al*.

Supplementary Note 1. Characterization of the population

A set of 268 diverse germplasm lines including 100 *indica*, 50 *aus* and 118 *japanica*, originated from geographic regions around the world, were genotyped as the mini-core collections of rice for salt tolerance evaluation (Supplementary Fig. 3a). The percentage of the entries in eastern China was 33.58%, followed by south-west China 19.40%, south China 8.58%, north China 7.84%, central China 7.09%, northeast China 7.84%, northwest China 2.24%, and other countries (India, Japan, Korea and Southeastern Asia) 13.83% (Supplementary Fig. 3a, Supplementary Data 2). We observed eight salt tolerant traits of shoot height (SH), shoot fresh weight (SFW), shoot dry weight (SDW), water content (WC), shoot Na^+ concentration (SNC), shoot K^+ concentration (SKC), shoot Na^+/K^+ concentration ratio (SNKR), and survival rate (SR) at 10 days of recovering. The phenotypic data showed significant segregation under control and salt treatment condition respectively. Histograms of normalized phenotypic values were evaluated for the seven traits and survival rate (Supplementary Fig. 1). We calculated survival rate and ratio of the other seven traits under salt treat/control for further association analysis (Supplementary Table 1).

To capture all common variants in rice mini-core accessions, we employed two-stage genotyping strategy. We generated a list of SNVs using low-coverage sequencing and then imputed genotype probabilities at these sites using near 3000 re-sequenced panel as reference.

We obtained a mean coverage of 0.06× per line for the 268 rice accessions through genotyping-by-sequencing and identified a total of 81,615 polymorphic SNVs in the all-sequence data that segregated in the population and the filtering criteria (minor allele frequency > 5%, missing rate < 0,9) were used to obtain 40889 polymorphic SNVs (online methods) (Supplementary Fig. 2). We then imputed genotype dosages at these sites using rice3k re-sequenced panel as references. Following stringent quality control after imputation, we retained 2,535,266 high-quality imputed SNVs for subsequent analysis. Accuracy at these sites was very high: the mean SNV-wise correlation (r^2) with was 0.98 after quality control. We annotated the high-quality imputed SNVs using the rice reference assembly and identified 82,262 SNVs positions in protein-coding sequence causing amino acid changes in individual genes (nonsynonymous substitutions) and 89,539 SNVs that did not cause such changes (synonymous substitutions) (Supplementary Table 3). The distribution of variants across the genome was highly non-uniform (Supplementary Fig. 2). Relatively low SNV polymorphisms were observed in the regions highlighted in green bars (Supplementary Fig. 2), which could be due to stringent recombination restrictions, or an extreme bottleneck in the founding of *aus* line.

Using the imputed SNVs, we performed population structure analysis to identify clusters of genetically similar accessions. Thus, the optimal subpopulations from STRUCTURE analysis numbered five groups (G): G1, G2, G3, G4 and G5 with each containing 119, 29, 9, 65 and 46 accessions, respectively (Fig. 1D, Supplementary Fig. 3b, Supplementary Data 2). All *japonica* accessions are clustered into G1. The *indica* lines were

divided into G4 and G5 whereas *aus* lines are primarily clustered into G2 and G3 as revealed also by phylogenetic tree (Fig. 1E). The score plot of principal components showed distribution with distinct clusters, indicating that the varieties represented a structured population (Fig. 1F).

Supplementary Note 2. SNV annotation

After stringent QC, we generated a final set of 2,535,266 high-quality imputed SNVs which all were annotated by SnpEff 3.0. Among the nucleotide variants, there is the largest number of SNVs in the chromosome 1 (321,642) whereas chromosome 9 has the least number of SNVs (156,150) (Supplementary Fig. 4a). We observed a strong linear relationship between the number of SNVs and chromosome length ($R^2=0.8084$, p=6.94×10^{-5}, Supplementary Fig. 4b). 85,520 SNVs induced nonsynonymous substitutions (Supplementary Fig. 4c, Supplementary Table 3).

Supplementary Note 3. Validation of rapid gene identification using GWAS

Using a mixed linear model, we performed GWAS for salt-tolerance-related traits to assess the potential of our GWAS design for causal gene identification. We detected 83 loci exceeding a significant threshold ($-\log_{10}P \geqslant 4$) performed in the present study (Supplementary Data 3). We focused on nine of these loci, including 6 peaks located on chromosomes 1, 3 and 9 with positions correlating with previously reported salt-tolerance-related genes *OsHKT1;5*(*OsSKC1)*, *OsHKT2;3*, *OsWRKY13*, *OsHAK2*, *OsHAK5*, *OsHAP2E* and *OsDSG1*.

On chromosome 1, there was a peak that mapped close to *OsSKC1* (*OsHKT1;5*) (Supplementary Fig. 3c). We estimated a candidate region from 11.29 Mb to 11.59 Mb by using pairwise LD correlations ($r^2 \geqslant 0.6$) (Supplementary Fig. 6a). The candidate region on chromosome 1 contained 977 polymorphisms. Through SNP annotation analysis, there were 10 polymorphisms assigned to missense-variant in a 104 kb LD block associated with salt tolerance harboring five genes. Further gene annotation showed that only one gene annotated as *OsSKC1* that was reported as a salt tolerance genes[1], whereas the remaining four were annotated as expressed protein (Supplementary Table 4). Two haplotypes of *OsSKC1* gene in the core population showed differences in salt tolerance (Supplementary Fig. 6b). The varieties carrying haplotype A showed stronger salt tolerance compared to varieties carrying haplotype B (Supplementary Fig. 6c).

Another peak on chromosome 1 was mapped close to *OsHKT2;3* (Fig. 1G, Supplementary Fig. 3c). The candidate region was predicted to map from 19.00 to 19.35 Mb (350 kb), and contained 3223 polymorphisms (Supplementary Fig. 7a). Among these, there were 51 polymorphisms assigned to missense-variant and mapped to 17 genes. Further gene annotation showed that these genes were annotated as enzyme (six),

hypothetical protein (six), expressed protein (two), DUF family protein (two) or ionic transporter (one) (Supplementary Data 4). We focused on *LOC_Os01g34850* (*OsHKT2;3*), which was annotated as a high-affinity potassium uptake transporter. The varieties contained four haplotypes (Supplementary Fig. 7b). The haplotypes associations with the salt tolerance phenotypes suggest that haplotype A is a functional haplotype for salt tolerance (Supplementary Fig. 7c).

The third peak on chromosome 1 was mapped closing to *OsWRKY13* (Supplementary Fig. 3c). The candidate region from 31.38 to 31.65 Mb (270 kb) contained 2430 polymorphisms (Supplementary Fig. 8a). Among these, there were 48 polymorphisms assigned to missense-variant containing 15 genes. Further gene annotation showed that these genes were annotated as enzyme (two), hypothetical protein (five), expressed protein (four), zinc-containing protein (two) and transcription factor (two) (Supplementary Data 6). We focused on *LOC_Os01g54600* (*OsWRKY13*)[2], which was reported down expression under salt treatment. The varieties contained three haplotypes (Supplementary Fig. 8b). The varieties carrying haplotype C showed higher shoots dry weights and lower shoots Na^+ concentrations. The haplotypes associations with the salt tolerance phenotypes suggest that haplotype C is a functional haplotype for salt tolerance (Supplementary Fig. 8c).

The fourth peak on chromosome 1 was mapped closing to *OsHAK5* and *OsHAK2* (Supplementary Fig. 3c). We estimated a candidate region from 40.63 Mb to 41.14 Mb (510 kb), and contained 2549 polymorphisms (Supplementary Fig. 9a). Among these, there were 77 polymorphisms assigned to missense-variant and mapped to 34 genes. Further gene annotation showed that these genes were annotated as enzymes (nine), hypothetical protein (seven), expressed protein (nine), protein kinase (five), transcription factor (two) and ionic transporter (two) (Supplementary Data 5). We focused on the two ionic transporter genes *LOC_Os01g70490* (*OsHAK5*) and *LOC_Os01g70940* (*OsHAK2*)[3], which were previously reported genes underlying salt tolerance. Haplotype analysis showed that three haplotypes were detected in both genes (Supplementary Fig. 9b and d) with highly significant differences in ST-SR and ST-WC in response to salt stress. Haplotype A with higher survival rates (SR) and water contents (WC) is a functional haplotype for salt tolerance in these genes (Supplementary Fig. 9c and e).

On chromosome 3, there was a peak that mapped close to *OsHAP2E* (Supplementary Fig. 3c). The candidate region was predicted to map from 16.66 Mb to 17.02 Mb (360 kb), and contained 3317 polymorphisms (Supplementary Fig. 10a). Through SNP annotation analysis, there were 25 polymorphisms assigned to missense-variant and mapped to 10 genes. Further gene annotation showed that most of genes were annotated as enzyme (three), hypothetical protein (two), expressed protein (four) and only one gene annotated as transcription factor (Supplementary Table 5). We focused on the nuclear transcription factor Y subunit gene *LOC_Os03g29760* (*OsHAP2E*)[4], which was reported associate with salt tolerance. There were four haplotypes of OsHAP2E gene in the core population that showed differences in salt tolerance (Supplementary Fig. 10b). The varieties carrying haplotype B showed more salt tolerance phenotypes than other haplotypes (Supplementary Fig. 10c).

The peak on chromosome 9 was mapped close to *OsDSG1* (Supplementary Fig. 3c). The candidate region was predicted to map from 15.71 to 16.34 Mb (630 kb), that contained 5026 polymorphisms (Supplementary Fig. 11a). Through SNP annotation analysis, these polymorphisms were mapped to 40 genes (Supplementary Data 7). Among these genes, we focused on *LOC_Os09g26400* (*OsDSG1*)[5], which was reported negatively regulated to salt tolerance. Further haplotype analysis showed that the varieties contained three haplotypes (Supplementary Fig. 11b) with highly significant differences in ST-SFW and ST-SH in response to salt stress. The haplotype associations with the salt tolerance phenotypes suggested that haplotype C is the functional haplotype for salt tolerance (Supplementary Fig. 11c).

With respect to the peak loci on chromosome 8, which exhibited associations with ST-SFW (Salt tolerance- shoots fresh weights) and ST-SDW (Salt tolerance- shoots dry weights) (Supplementary Fig. 3c). The candidate region was predicted to map from 28.11 to 28.32 Mb (210 kb), and contained 1412 polymorphisms (Supplementary Fig. 12a). Through SNP annotation analysis, there were 53 polymorphisms assigned to missense-variant and mapped to 15 genes. Further gene annotation showed that most of genes were annotated as unknown function protein (two), expressed protein (six), transport protein (two), enzyme (three), protein kinase (one) and transcription factor (one) (Supplementary Data 8). Further haplotype analysis for the protein kinase and transcription factor, no significant differences were detected (Supplementary Fig. 14 c, b and d). In addition, the remaining three enzyme genes showed that *LOC_Os08g44840* and *LOC_Os08g44860* genes showed extremely significant differences in ST-SFW (Salt tolerance- shoots fresh weights) and ST-SDW (Salt tolerance- shoots dry weights) (Supplementary Fig. 12c, Supplementary Fig. 14 c). The SNP at genomic locus bp 28164360 associated with salt tolerance traits located in *LOC_Os08g44840*, which encodes a BAHD acetyltransferase. Here we found that *BAHD* associated with salt tolerance in rice.

Supplementary Note 4. Prioritization of two candidate genes

We applied the same strategy to loci associated with salt-tolerance-related trait, which have not been reported previously (i.e., chromosomes 3, 5 and 8) (Fig. 1G, Supplementary Fig. 3c). With respect to the locus on chromosome 5, the candidate region was predicted to map from 16.12 to 16.15 Mb (30 kb), and included 323 polymorphisms (Fig. 2A). Through SNP annotation analysis, most of these were annotated as upstream or downstream gene variants except for one polymorphism assigned to 5'UTR mapped to *LOC_Os05g27730* (*OsWRKY53*), a WRKY transcription factor. *OsWRKY53* previously reported as a modulator in regulating grain size in rice[6], and positively regulates brassinosteroid signaling and plant architecture[7], whereas the function of its rice salt tolerance was unknown. Phylogenetic relationship between WRKYs from rice cultivar with the other salt related WRKYs from different plants. *OsWRKY53* was not genetically related to the *OsWRKY13* gene (Supplementary Fig. 15a).

We then focused on the peak on chromosome 3, which exhibited associations with survival rates (SR). LD analysis showed that three LD blocks exist in the peak. We estimated the candidate region to be 6.45−6.87 Mb (420 kb) about the second LD block containing the highest peak (Fig. 4A). We assigned 46 polymorphisms assigned to missense-variant and mapped to 20 genes. Most of these genes (16 of 20) were annotated as either enzyme (four), hypothetical protein (four) or putative expressed protein (eight) expressed protein, whereas the remaining four were annotated as protein kinase (three) or cation efflux protein (one) (Supplementary Data 9). *LOC_Os03g12530* (*OsMTP8.1*) was previously reported as a Mn^{2+} specific transporter and no effect on other metal ions[8]. Further haplotype analysis for the remaining four genes showed that only *LOC_Os03g12390* gene showed extremely significant differences in ST-SR (Salt tolerance-survival rate), whereas the remaining three showed no significant differences (Supplementary Fig. 13). In addition, *LOC_Os03g12390* (*OsMKK10.2*) encodes mitogen activated protein kinase, that could enhance rice disease resistance and drought tolerance[9]. Phylogenetic relationship between *OsMKK10.2* from other related *MAPKKs* from different plants. *OsMKK10.2* was not genetically related to the other gene family members (Supplementary Fig. 15b). The function of *OsMKK10.2* on salt tolerance remains unknown.

Supplementary Note 5. Xylem parenchyma cell Na^+ flux analysis

For xylem parenchyma cell Na^+ flux analysis, seven-day-old rice seedlings were treated using 140 mM NaCl for 24 hours. Primary root was severed from the root-rhizome junction and fully balanced in measuring solution (0.1 mM KCl, 0.1 mM $CaCl_2$, 0.1 mM $MgCl_2$, 0.5 mM NaCl, 0.3 mM MES, 0.2 mM Na_2SO_4, pH=6) for at least 20 mins. The sample was fixed in culture dish by resin block and filter paper with fresh measuring solution. Micro-electrode was placed on the position of parenchyma cell to detect the Na^+ flux (Supplementary Fig. 16e).

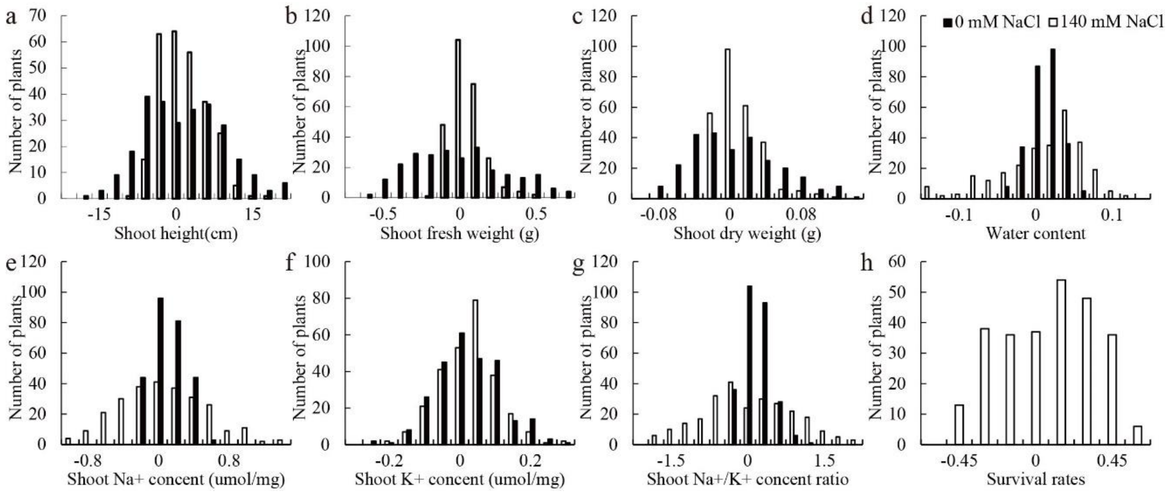

Supplementary Figure 1. Phenotypic diversity of 268 rice varieties. Histograms of zero mean normalized phenotypic values of shoot height (a), shoot fresh weight (b), shoot dry weight (c), water content (d), shoot Na^+ concentration (e), shoot K^+ concentration (f), shoot Na^+/K^+ concentration ratio (g), and survival rate (h). Black and white bars represented the data from phenotyping performed under control and salt treat condition respectively.

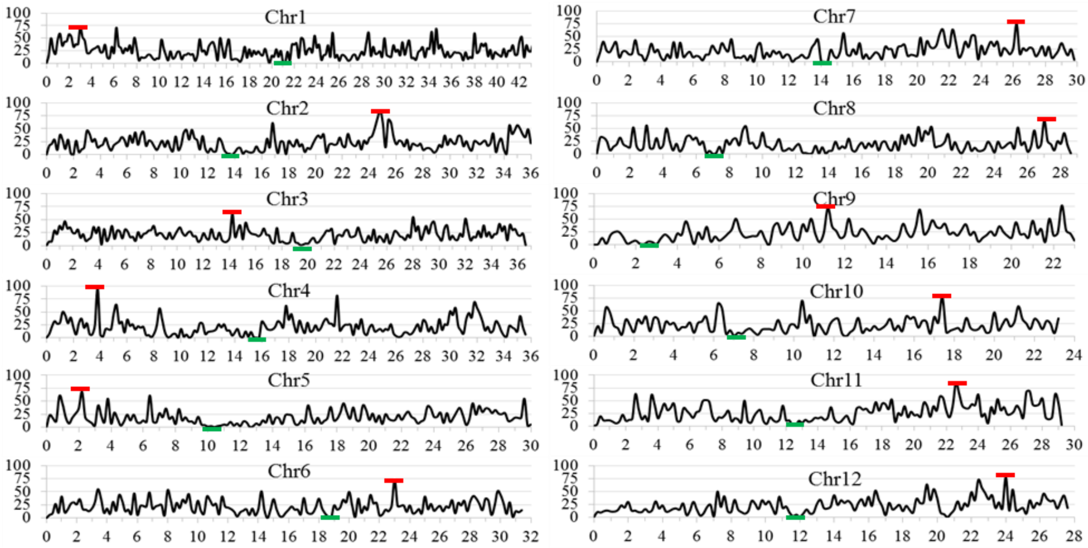

Supplementary Figure 2. Dispersal of RAD clusters with 40,889 SNPs/InDels across the 12 chromosomes of rice. The x-axis showed the pseudomolecule position in which each tick-mark was a megabase. The y-axis showed the number of SNP at 200 kb intervals. Regions that showed low SNP frequencies were noted with green blocks, and regions of higher than 75 SNPs /200kb frequencies were noted with red blocks.

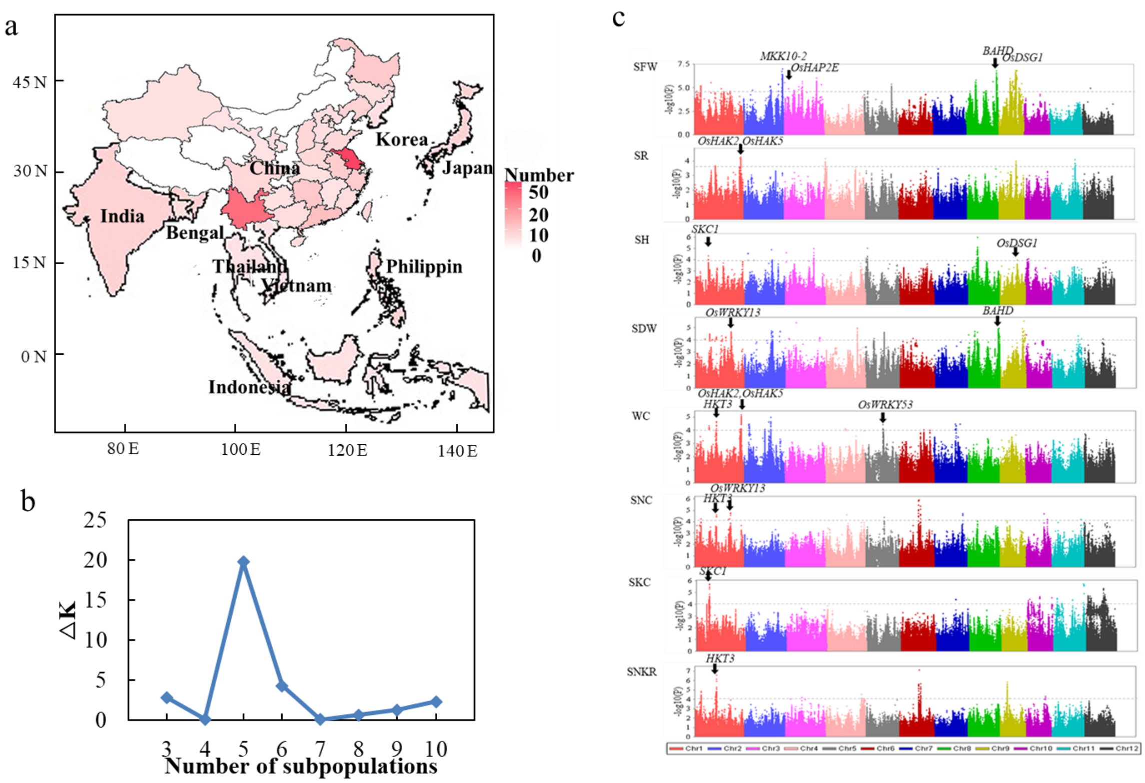

Supplementary Figure 3. STRUCTURE analysis. (a) Location of sampled landraces worldwide. The color represented the landrace number in the region. (b) The ΔK value was much higher for the model parameter K = 5 than for other values of K. (c) Overview of MLM. Scale: −log10 of *P* value of markers. Manhattan plots for SFW, SR, SH, SDW, WC, SNC, SKC and SNKR. Mixed linear model was used to calculate *P* value.

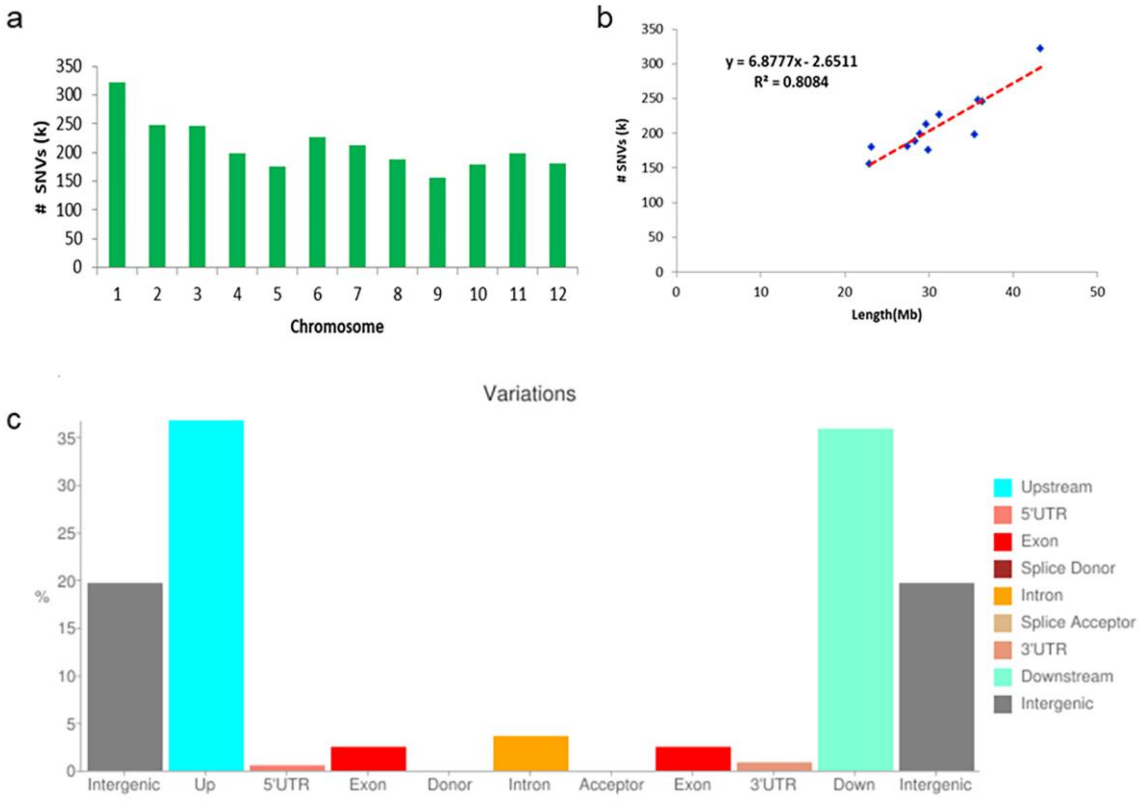

Supplementary Figure 4. Annotation of SNVs. (a) Distribution of SNVs by chromosome (b) SNVs explained by chromosome (c) Distribution of SNVs by regions.

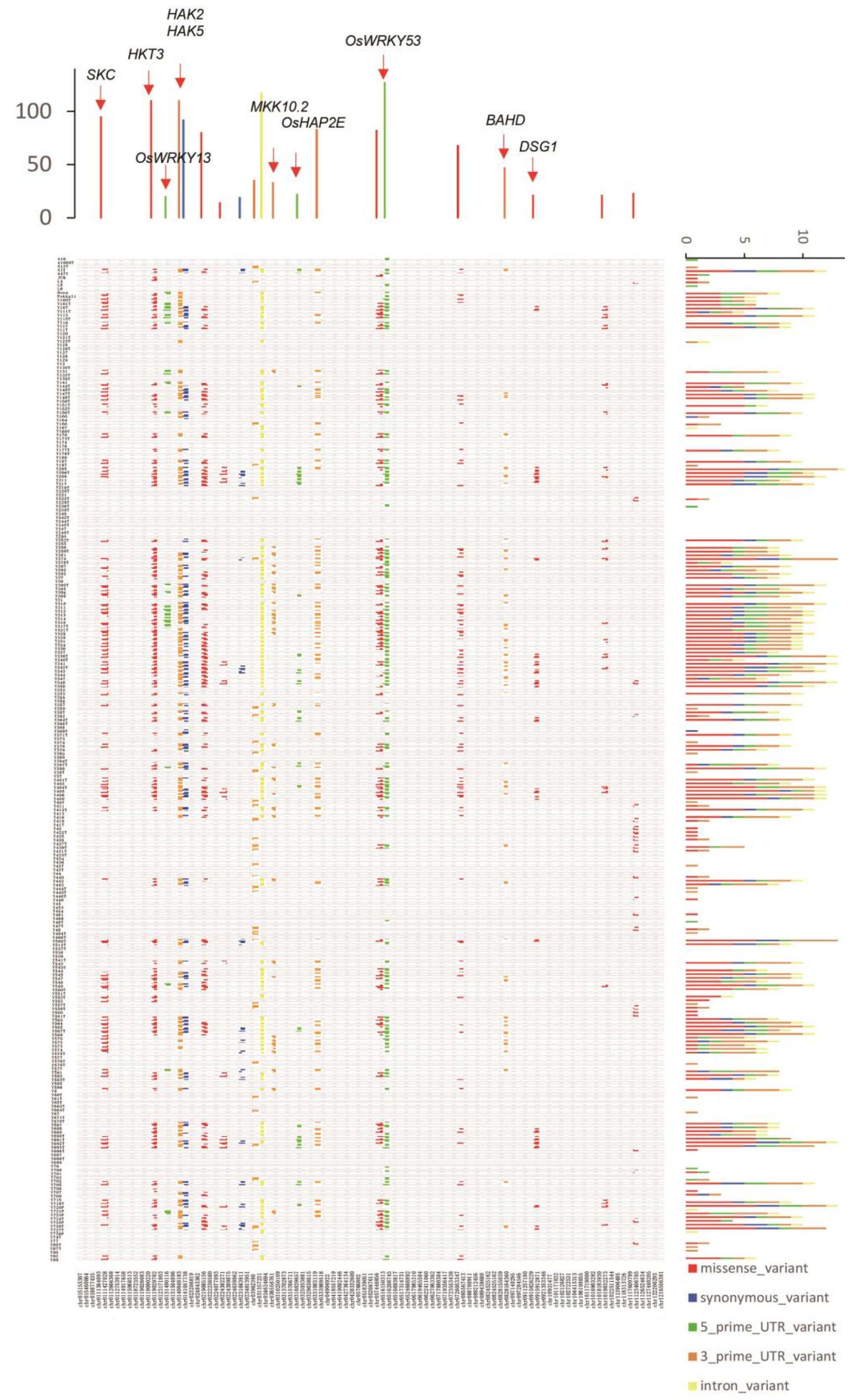

Supplementary Figure 5. Heatmap of the 19 loci associated with salt tolerance. Horizontal direction indicated histogram of genomic regions. Vertical direction showed histogram of accessions in in left side, and the number of variations in different accessions in the right side.

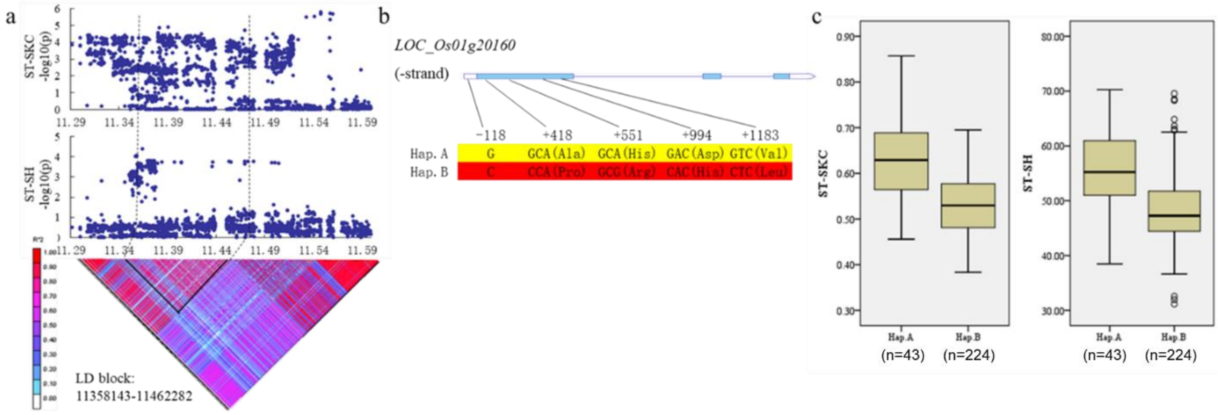

Supplementary Figure 6. Detailed analyses of the peak for ST-SH and ST-SKC on chromosome 1. (a) Local Manhattan plot (top) and LD heatmap (bottom) surrounding the peak on chromosome 1. (b) Exon-intron structure of *LOC_Os01g20160* (*HKT1;5*) and DNA polymorphisms in this gene. (c) Boxplots for ST-SKC (left) and ST-SH (right) based on the haplotypes (Hap) for *LOC_Os01g20160* (*HKT1;5*). Box edges represented the 0.25 quantile and 0.75 quantile with the median values shown by bold lines. Whiskers extended to data no more than 1.5 times the interquartile range and remaining data were indicated by dots.

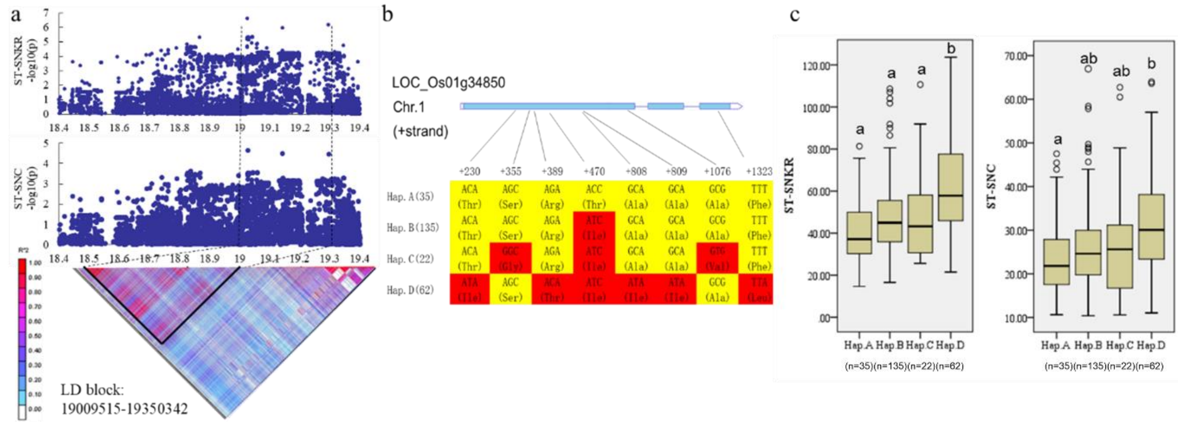

Supplementary Figure 7. Detailed analyses of the peak for ST-SNC and ST-SNKR on chromosome 1. (a) Local Manhattan plot (top) and LD heatmap (bottom) surrounding the peak on chromosome 1. **(b)** Exon-intron structure of *OsHKT2;3* and DNA polymorphism in that gene. **(c)** Boxplots for ST-SNC (left) and ST-SNKR (right) based on the haplotypes (Hap) for *LOC_Os01g34850* (*OsHKT2;3)*. Box edges represented the 0.25 quantile and 0.75 quantile with the median values shown by bold lines. Whiskers extended to data no more than 1.5 times the interquartile range and remaining data were indicated by dots. Letters indicated significant differences among different treatments ($P < 0.05$; Tukey's test).

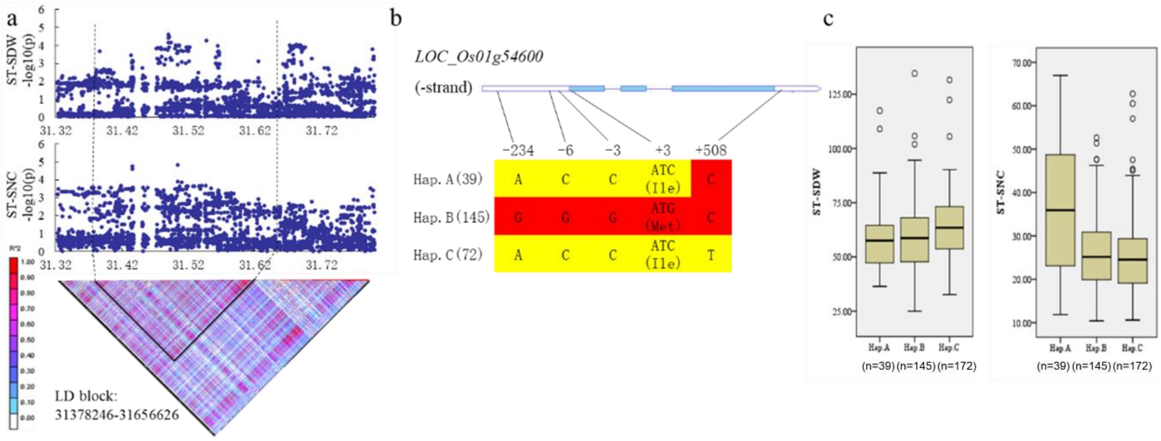

Supplementary Figure 8. Detailed analyses of the peak for ST-SDW and ST-SNC on chromosome 1. (a) Local Manhattan plot (top) and LD heatmap (bottom) surrounding the peak on chromosome 1. (b) Exon-intron structure of *LOC_Os01g54600* (*OsWRKY13*) and DNA polymorphisms in this gene. (c) Boxplots for ST-SDW (left) and ST-SNC (right) based on the haplotypes (Hap) for *LOC_Os01g54600* (*OsWRKY13*). Box edges represented the 0.25 quantile and 0.75 quantile with the median values shown by bold lines. Whiskers extended to data no more than 1.5 times the interquartile range, and remaining data were indicated by dots.

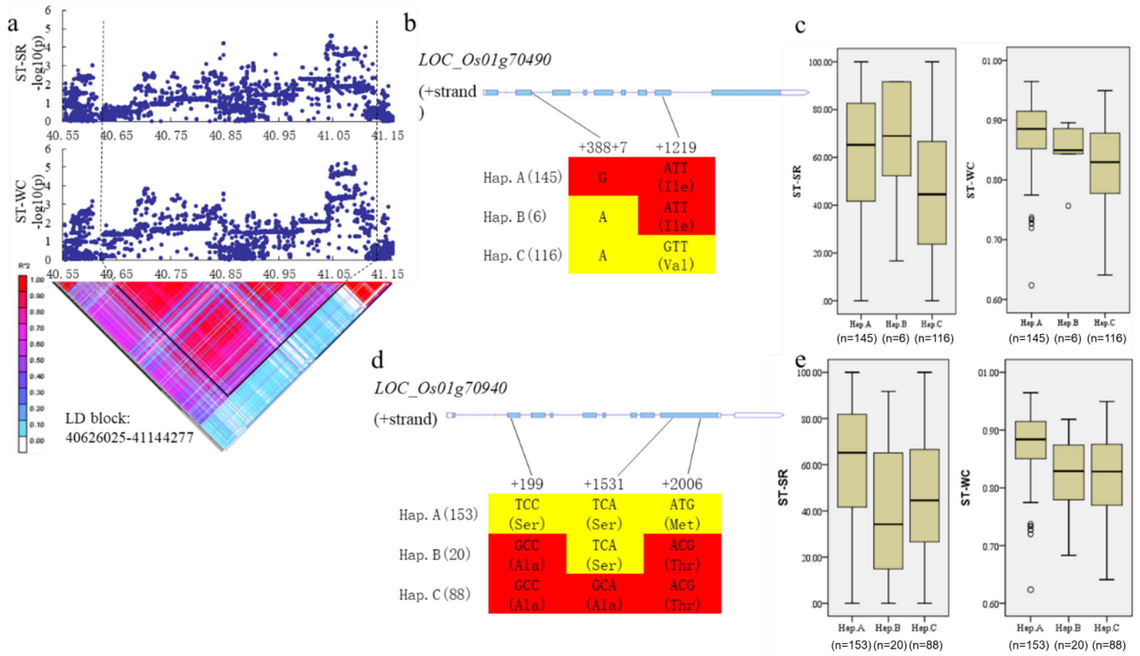

Supplementary Figure 9. Detailed analyses of the peak for ST-SR and ST-WC on chromosome 1. (a) Local Manhattan plot (top) and LD heatmap (bottom) surrounding the peak on chromosome 1. (b) Exon-intron structure of *LOC_Os01g70490* (*OsHAK5*) and DNA polymorphisms in this gene. (c) Boxplots for ST-SR (left) and ST-WC (right) based on the haplotypes (Hap) for *LOC_Os01g70490* (*OsHAK5*). (d) Exon-intron structure of *LOC_Os01g70940* (*OsHAK2*) and DNA polymorphisms in this gene. (e) Boxplots for ST-SR (left) and ST-WC (right) based on the haplotypes (Hap) for *LOC_Os01g70940* (*OsHAK2*). Box edges represented the 0.25 quantile and 0.75 quantile with the median values shown by bold lines. Whiskers extended to data no more than 1.5 times the interquartile range, and remaining data were indicated by dots.

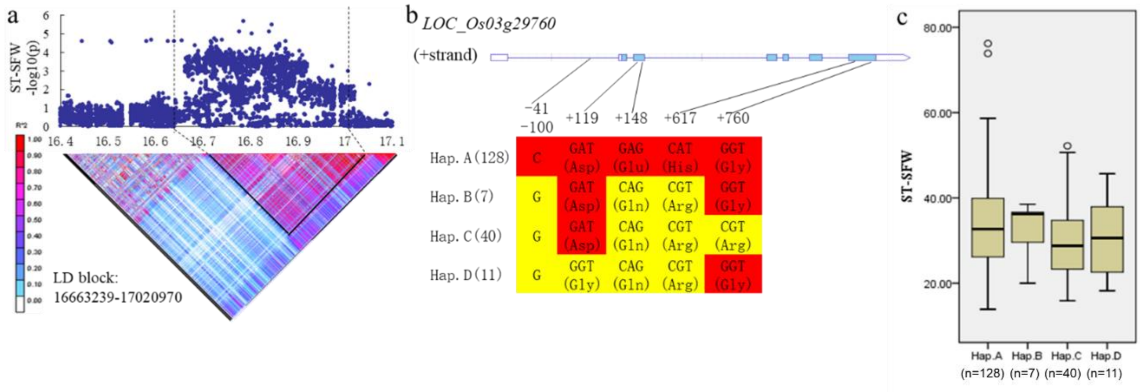

Supplementary Figure 10. Detailed analyses of the peak for ST-SFW on chromosome 3. (a) Local Manhattan plot (top) and LD heatmap (bottom) surrounding the peak on chromosome 3. (b) Exon-intron structure of *LOC_Os03g29760* (*OsHAP2E*) and DNA polymorphisms in this gene. (c) Boxplots for ST-SFW based on the haplotypes (Hap) for *LOC_Os03g29760* (*OsHAP2E*). Box edges represented the 0.25 quantile and 0.75 quantile with the median values shown by bold lines. Whiskers extended to data no more than 1.5 times the interquartile range, and remaining data were indicated by dots.

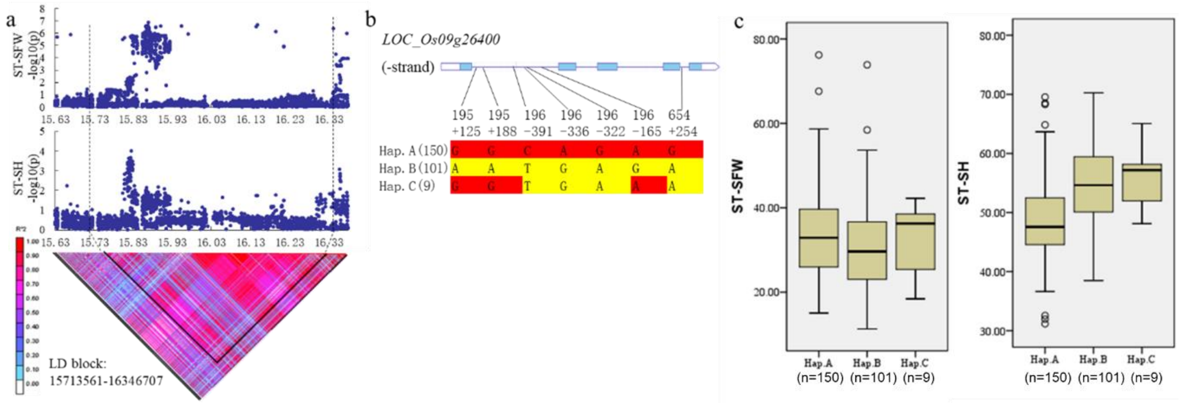

Supplementary Figure 11. Detailed analyses of the peak for ST-SFW and ST-SH on chromosome 9. (a) Local Manhattan plot (top) and LD heatmap (bottom) surrounding the peak on chromosome 9. (b) Exon-intron structure of *LOC_Os09g26400* (*OsDSG1*) and DNA polymorphisms in this gene. (c) Boxplots for ST-SFW (left) and ST-SH (right) based on the haplotypes (Hap) for *LOC_Os09g26400* (*OsDSG1*). Box edges represented the 0.25 quantile and 0.75 quantile with the median values shown by bold lines. Whiskers extended to data no more than 1.5 times the interquartile range, and remaining data were indicated by dots.

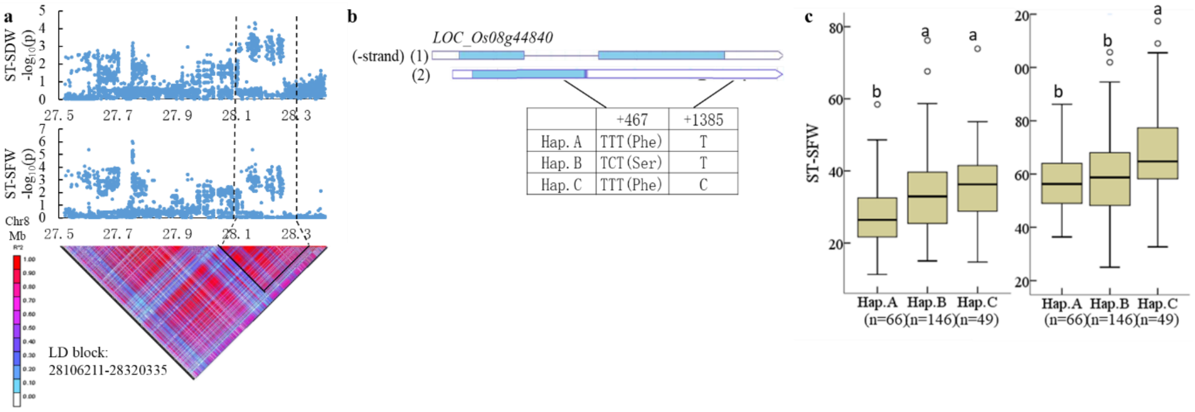

Supplementary Figure 12. Identification and characterization of the causal gene *OsBAHD*. (a) Local Manhattan plot (top) and LD heatmap (bottom) surrounding the peak on chromosome 8. (b) Exon-intron structure of *OsBAHD* and DNA polymorphisms in this gene. (c) Boxplots for ST-SFW (left) and ST-SDW (right) based on the *OsBAHD* haplotypes (Hap). Box edges represented the 0.25 and 0.75 quantiles with the median values shown by bold lines. Whiskers extended to data no more than 1.5 times the interquartile range, and remaining data were indicated by dots. Letters indicate significant differences among different treatments ($P < 0.05$; Tukey's test).

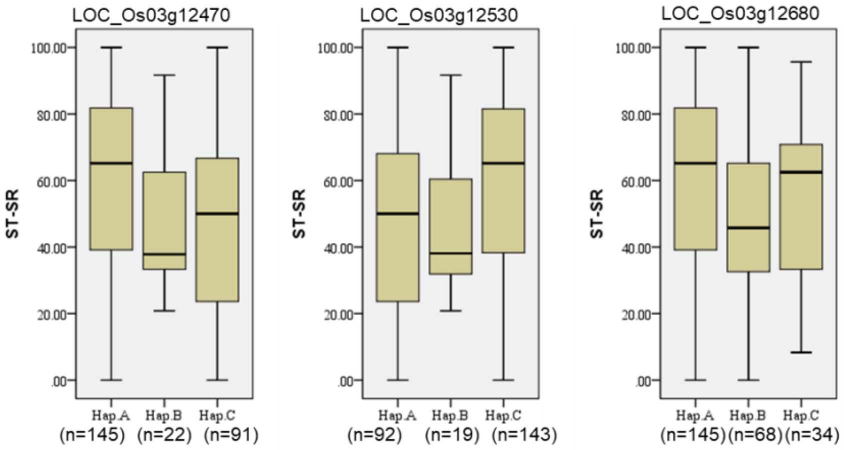

Supplementary Figure 13. Boxplots for ST-SR based on the haplotypes (Hap) of chromosome 3. Box edges represented the 0.25 and 0.75 quantiles with the median values shown by bold lines. Whiskers extended to data no more than 1.5 times the interquartile range.

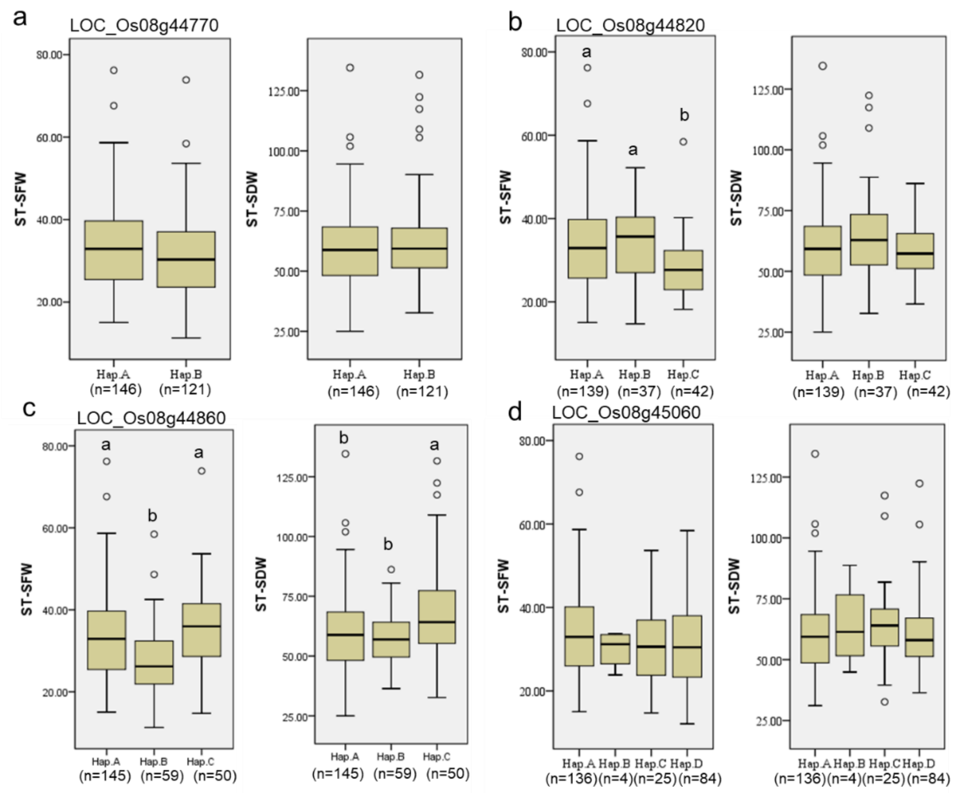

Supplementary Figure 14. Boxplots for ST-SFW and ST-SDW based on the haplotypes (Hap). (a) LOC_Os08g44770, (b) LOC_Os08g44820, (c) LOC_Os08g44860 and (d) LOC_Os08g45060 in chromosome 8. Box edges represented the 0.25 and 0.75 quantiles with the median values shown by bold lines. Whiskers extended to data no more than 1.5 times the interquartile range, and remaining data were indicated by dots. Differences between the haplotypes were statistically analyzed based on Turkey's test ($P < 0.01$).

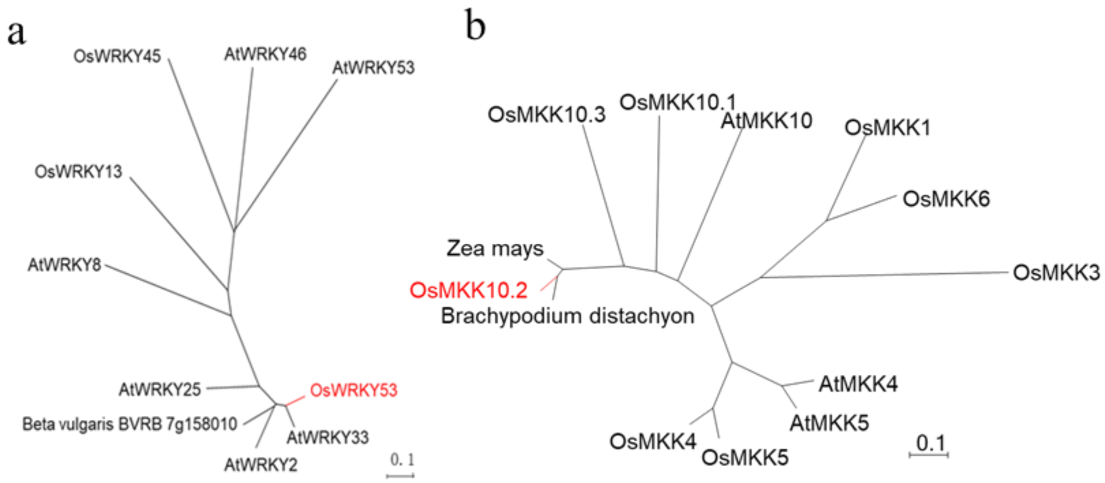

Supplementary Figure 15. Phylogenetic relationship between salt related genes from different plants. (a) Phylogenetic relationship between WRKYs from rice cultivar with the other salt related WRKYs from different plants. (b) Phylogenetic relationship between OsMKK10.2 from other related MAPKKs from different plants.

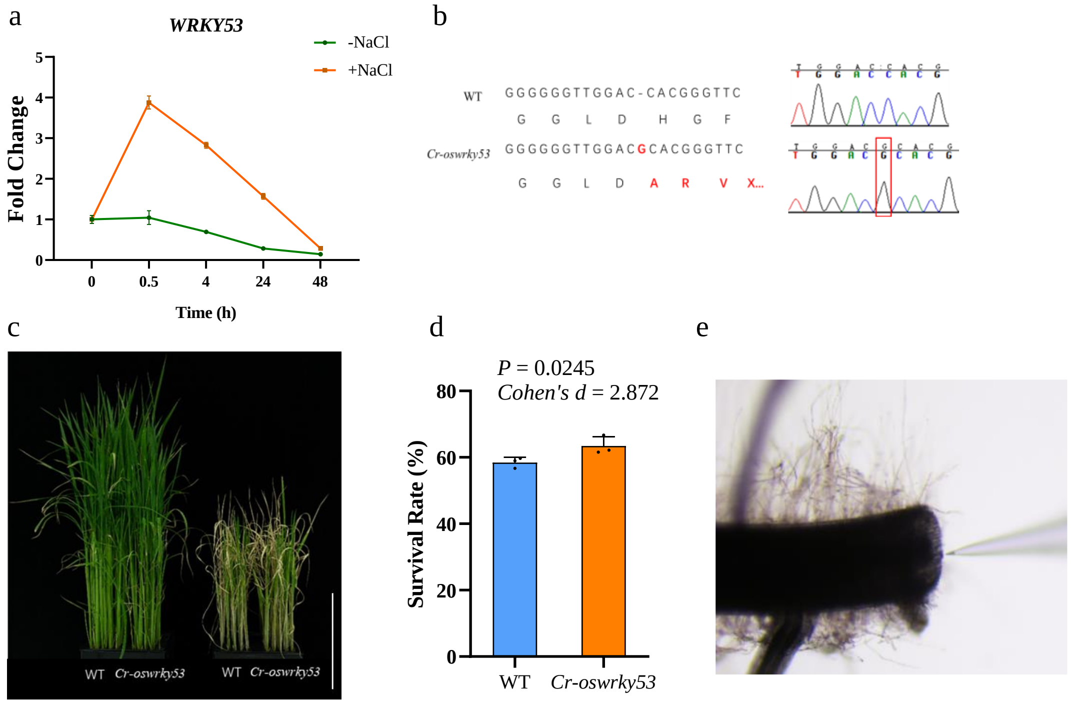

Supplementary Figure 16. *OsWRKY53* responded to salt stress. (a) Expression of *WRKY53* under control and salt treated (140mM NaCl). Data were presented as means ± SD, n = 3. (b) Schematic diagram of *WRKY53* in WT and *Cr-oswrky53*. (c) Phenotype of *WRKY53* knock-out line under 140mM NaCl treatment. Bar = 12cm. (d) Survival rates of WT and *Cr-oswrky53* under 140mM NaCl treatment. Thirty-two plants were used to determine the survival rate. Data were presented as means ± SD, n = 3. *P* values were calculated with two-sided Student's *t*-test. (e) Na^+ flux of root xylem parenchyma cell in rice seedling was measured via NMT analysis. Source data were provided as a Source Data file.

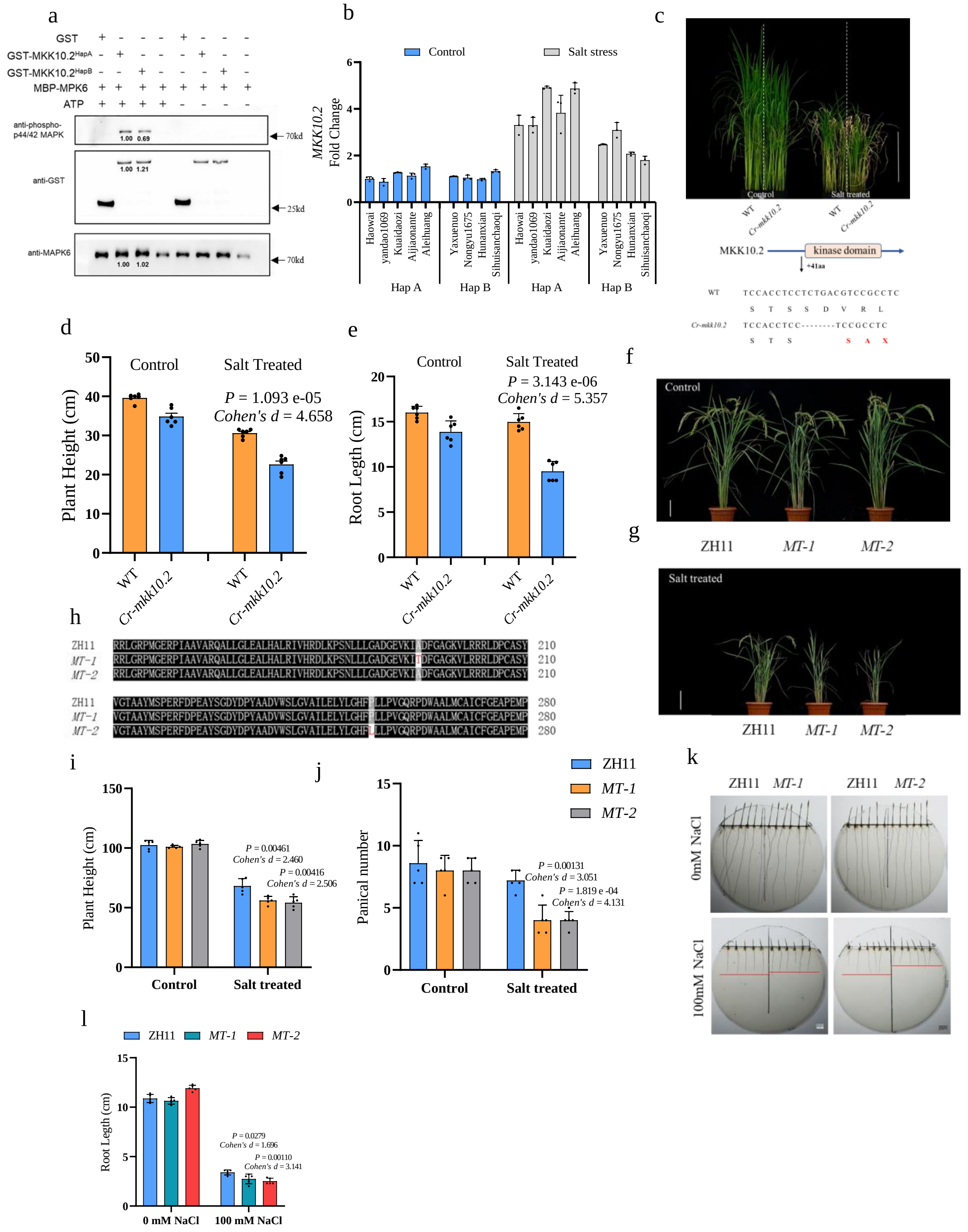

Supplementary Figure 17. *OsMKK10.2* responded to salt stress. (a) OsMKK10.2^{HapA} showed stronger kinase activity than OsMKK10.2^{HapB}. Purified proteins of GST-OsMKK10.2^{HapA}, GST- OsMKK10.2^{HapB} were used in vitro kinase assay. Anti-phospho-p44/42 MAPK was used to detect phosphorylated MPK6. Loading controls of GST-OsMKK10.2^{HapA}, GST- OsMKK10.2^{HapB}, MBP-MPK6 were detected using anti-GST, anti-MAPK6 respectively. *In vitro* kinase assay was repeated three times. (b) Expression analysis of candidate gene *MKK10.2* in haplotypes from different varieties. Data

represented means ±s.d. (n= 3). (c) Phenotype of *OsMKK10.2* knock-out line *Cr-mkk10.2* under 140mM NaCl treatment. Bar = 12cm. (d, e) Plant height (d) and root length (e) of WT and *Cr-mkk10.2* under 140mM NaCl treatment. The values were significantly different from that of the WT under 140 mM NaCl, n = 6. Data were presented as means ± SD. *P* values were calculated with two-sided Student's *t*-test. (f, g) Phenotypes of ZH11 (left) and *mkk10.2* mutant (right) in non-salt field (f) and salt stress field (g). Bar = 18cm. (h) Protein structures of ZH11 and two *mkk10.2* mutant plants. (i, j) Plant height (i) and panicle number (j) of ZH11 and *mkk10.2* mutant in non-salt field and salt stress field. The values were significantly different from that of the WT under salt stress field. Data were presented as means ± SD, n = 5. *P* values were calculated with two-sided Student's *t*-test. (k) Salt tolerance evaluation at germination of wild type ZH11 (left) and *mkk10.2* mutant (right) seeds. Pictures were taken 3 d post salt treatment, without (above panel) and with 100 mM NaCl (below panel). (l) Root length under salt stress conditions. Data were calculated 3 d post salt stress. The values were significantly different from that of the WT under 100 mM NaCl, n = 5, data were presented as means ± SD. *P* values were calculated with two-sided Student's *t*-test. Source data were provided as a Source Data file.

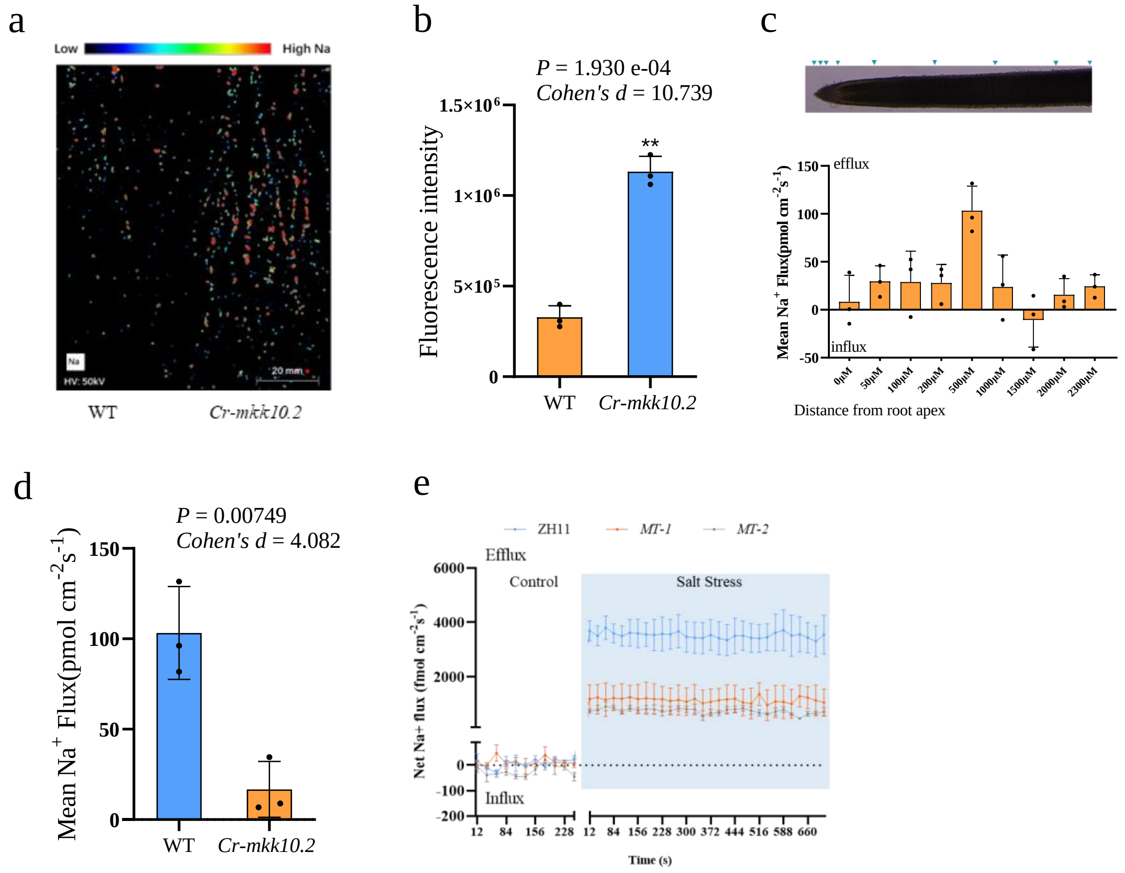

Supplementary Figure 18. MKK10.2 mediated rice salt tolerance through regulating ion homeostasis. (a) The image of μ-XRF in root of WT and *Cr-mkk10.2* under 140 mM NaCl, respectively. (b) Fluorescence intensity of μ-XRF scan in root of WT, *Cr-mkk10.2* under 140 mM NaCl (a). Data were presented as means ± SD, n = 3. *P* values were calculated with two-sided Student's *t*-test. (c) Net Na^+ efflux scan in different distance from root apex using non-injuring technique under 140mM NaCl treatment. (d) Mean Na^+ fluxes in 500 μm distance from root apex of WT and *Cr-mkk10.2*. For each line, three seedlings were used for Na^+ flux analysis. Data were presented as means ± SD. *P* values were calculated with two-sided Student's *t*-test. (e) Net Na^+ fluxes in 500 μm distance from root apex of *MT-1*, *MT-2* and WT plants measured using non-invasive micro-test technology. Data were presented as mean values ± SE, n = 3. Source data were provided as a Source Data file.

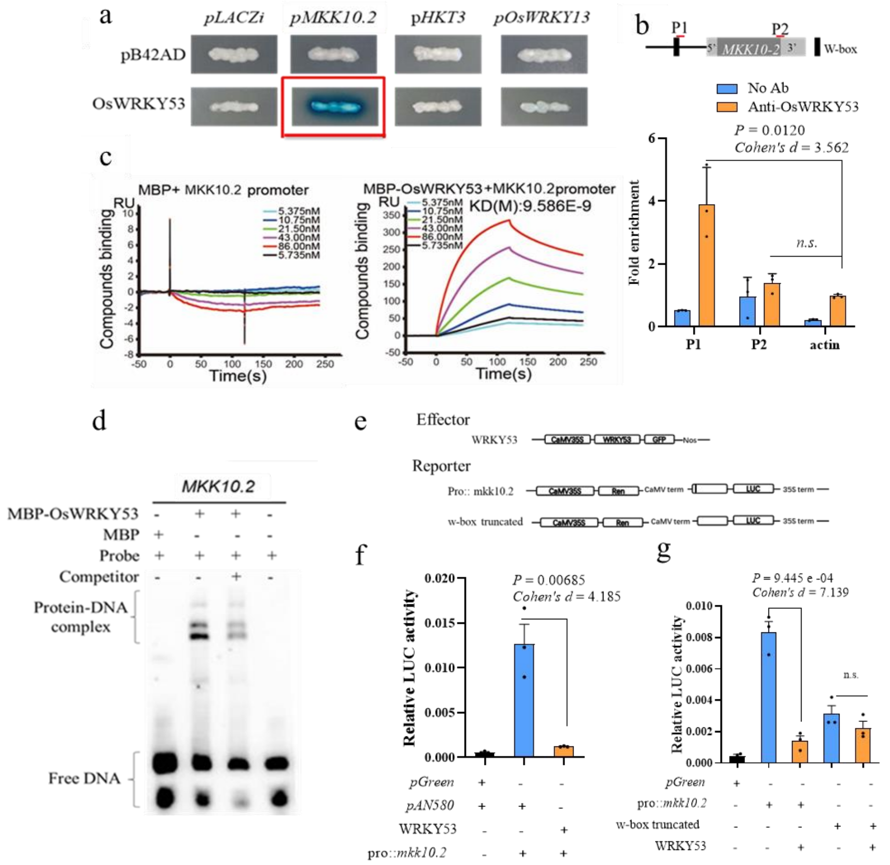

Supplementary Figure 19. Interactions between OsWRKY53 and *OsMKK10.2*. (a) Yeast one-hybrid assays to study interactions between transcription factors OsWRKY53 and target gene *OsMKK10.2*. (b) ChIP analysis of OsWRKY53 binding to the promoter of *MKK10.2* in vivo. Immunoprecipitation was performed with anti-OsWRKY53 antibody. The DNA fragment (red line) either containing or non-containing W-box motif in the promoter regions of the targets. RT-qPCR enrichment was calculated by normalizing to actin and to the total input of each sample. Data shown as means ± SD (n = 3). *P* values were calculated by two-sided Student's *t*-test. (c) OsWRKY53 binding to W-box elements in *OsMKK10.2* promoter in surface plasmon resonance (SPR) experiments by using Biacore T200 instrument. (d) DNA binding activity of OsWRKY53 protein on *OsMKK10.2* promoter fragments tested by EMSA assays. Brackets showed protein-DNA complex or free probe respectively. EMSA assay was repeated three times. MBP-OsWRKY53 fusion protein was expressed in *E. coli*. The probes were labeled with biotin. (e) Schematic representation of constructs using in *MKK10.2* promoter LUC activity measurement. (f) W-box element in *OsMKK10.2* promoter and transcriptional activation activity assay in rice protoplast. The values were significantly different from that of the control. Data were presented as means ± SD, n = 3. *P* values were calculated with two-sided Student's *t*-test. (g) The firefly luciferase gene driven by *MKK10.2* promoter and truncated *MKK10.2* promoter without w-box. Data were normalized to the internal control 35S::REN. The values were significantly different from that of the control. Data were presented as means ± SD, n = 3. *P* values were calculated with two-sided Student's *t*-test. Source data were provided as a Source Data file.

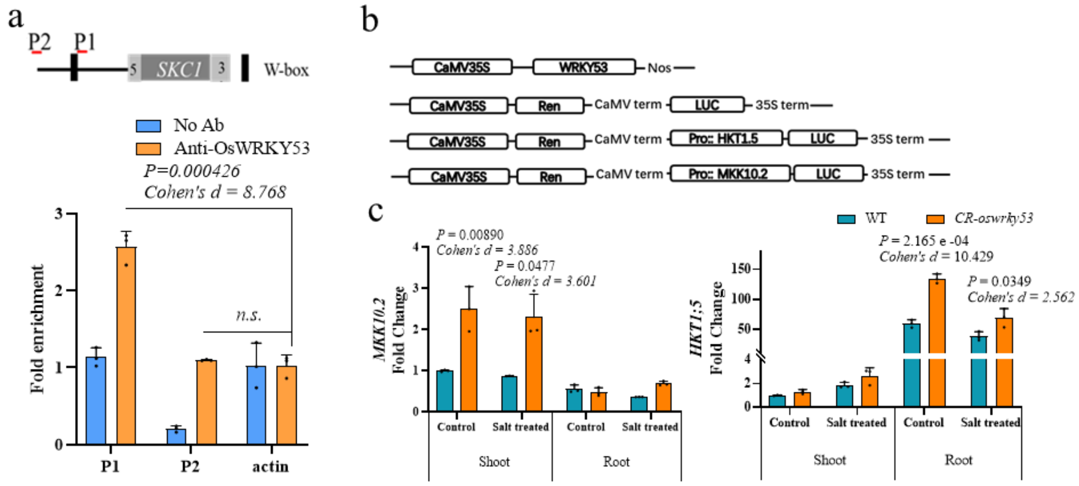

Supplementary Figure 20. Interactions between OsWRKY53 and *HKT1;5*. (a) ChIP analysis of OsWRKY53 binding to the promoter of *HKT1;5* in vivo. Immunoprecipitation was performed with anti-OsWRKY53 antibody. The DNA fragment (red line) either containing or non-containing W-box motif in the promoter region of the targets. RT-qPCR enrichment was calculated by normalizing to actin and to the total input of each sample. Data shown as means ± SD (n = 3). *P* values were calculated by two-sided Student's *t*-test. (b) Schematic diagrams of the effector and reporter constructs used for transient expression assay. (c) Expressions of *OsMKK10.2* and *OsHKT1;5* in *Cr-oswrky53* and WT roots and shoots under control and salt treated for 8 hours (140 mM NaCl). *OsActin* was used as endogenous control. Data shown as means ± SD (n = 3). *P* values were calculated by two-sided Student's *t*-test. Source data were provided as a Source Data file.

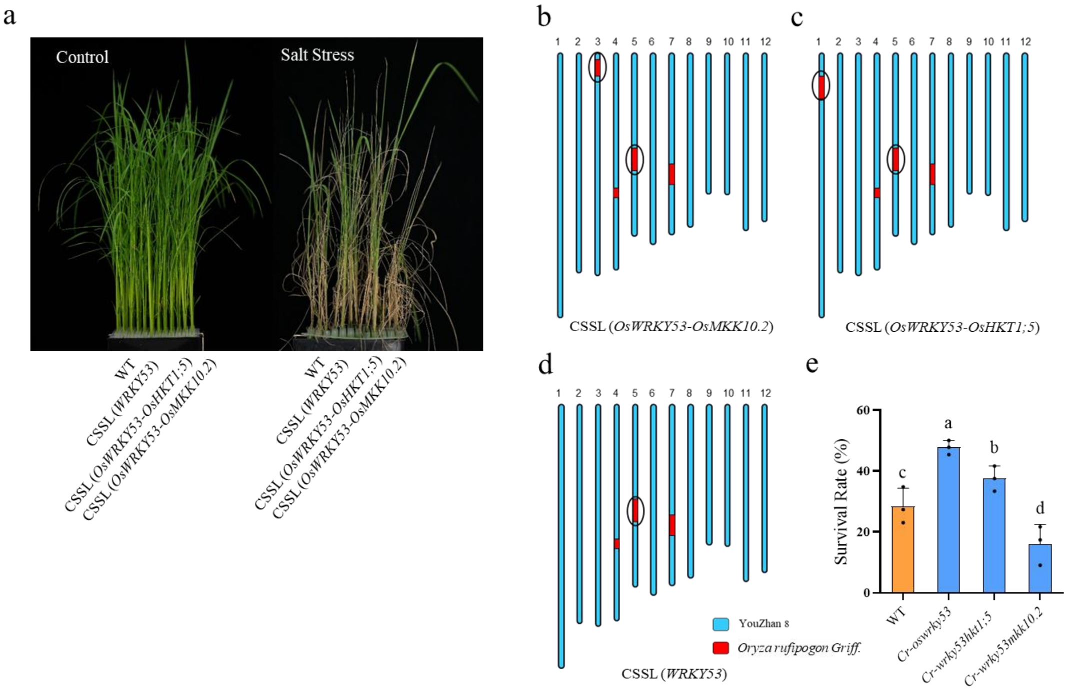

Supplementary Figure 21. Genetic interactions between *OsWRKY53-OsMKK10.2* and *OsWRKY53-OsHKT1;5*. (a) Phenotypes of CSSLs carrying *WRKY53MKK10.2* and *WRKY53HKT1;5* at 10 days post 140 mM NaCl treatment. (b-d) CSSLs with substitution segments containing *WRKY53MKK10.2* (b), *WRKY53HKT1;5* (c) and *WRKY53* (d) of *Oryza rufipogon Griff.* respectively. (e) Survival rates of *Cr-wrky53mkk10.2* and *Cr-wrky53hkt1;5* at 14 days post 140 mM NaCl treatment. Letters indicated significant differences among different treatments ($P < 0.05$; Tukey's test), n = 3. Source data were provided as a Source Data file.

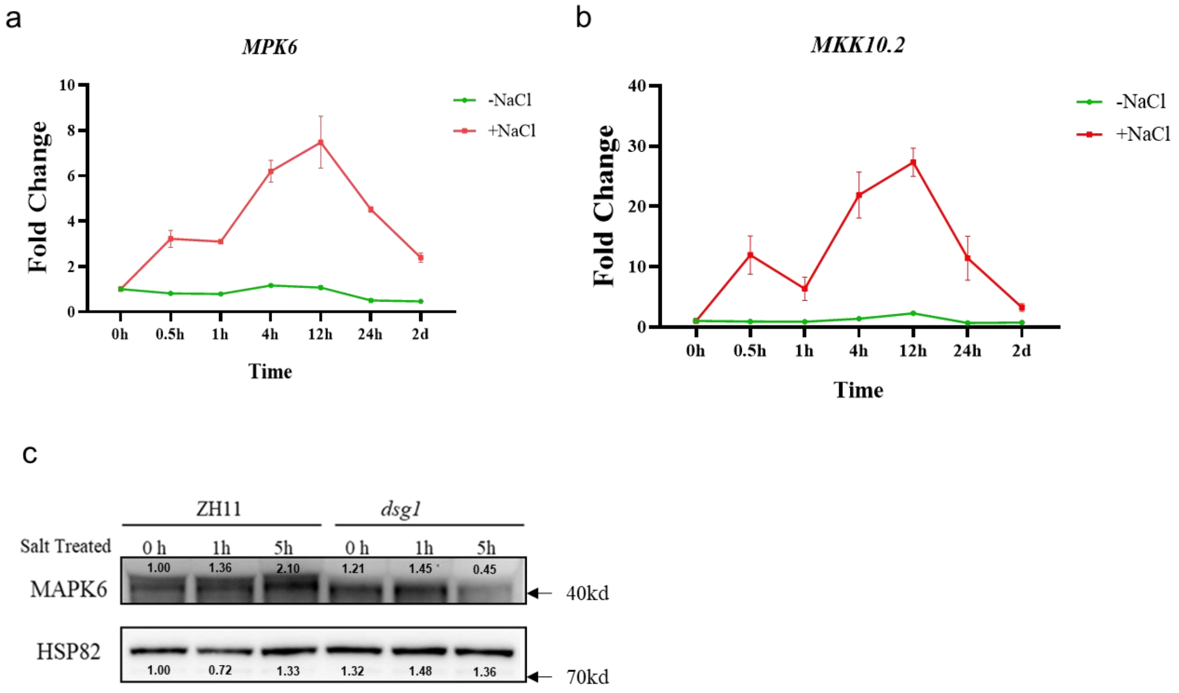

Supplementary Figure 22. *MPK6* in response to salt stress. (a, b) Expression levels of *MKK10.2* (a) and *MPK6* (b) in response to salt stress. Data were presented as means ± SD, n = 3. (c) Protein levels of MPK6 in wildtype and *dsg1* at 1h and 5h post salt treatment. Western bolt analysis was repeated three times. Source data were provided as a Source Data file.

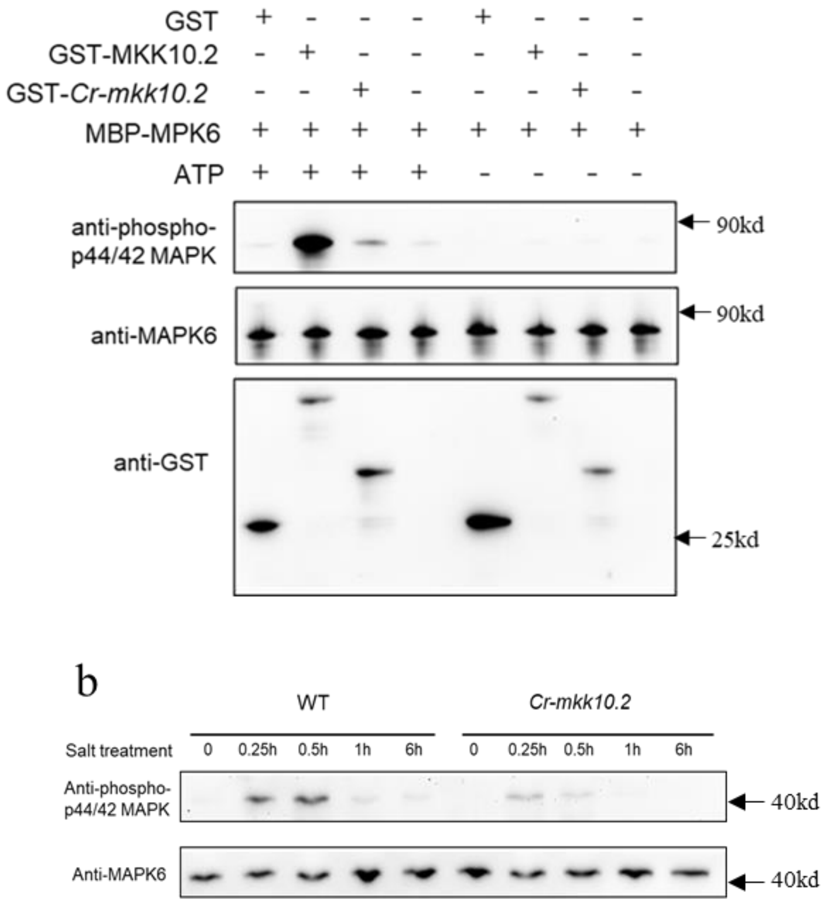

Supplementary Figure 23. MKK10.2 mediated rice salt tolerance through phosphorating MPK6. (a) *Cr-mkk10.2* failed to phosphorylate MPK6. Purified proteins of GST-MKK10.2, GST- *Cr-mkk10.2* were used in vitro kinase assay. Anti-phospho-p44/42 MAPK was used to detect phosphorylated MPK6. Loading controls of GST-MKK10.2, GST-*Cr-mkk10.2*, MBP-MPK6 were detected using anti-GST, anti-MAPK6 respectively. *In vitro* kinase assay was repeated three times. (b) Phosphorylation level of MPK6 in WT and *Cr-mkk10.2* during salt treatment. MPK6 was used as a control. Phosphorylation detection *in vivo* was repeated three times. Source data were provided as a Source Data file.

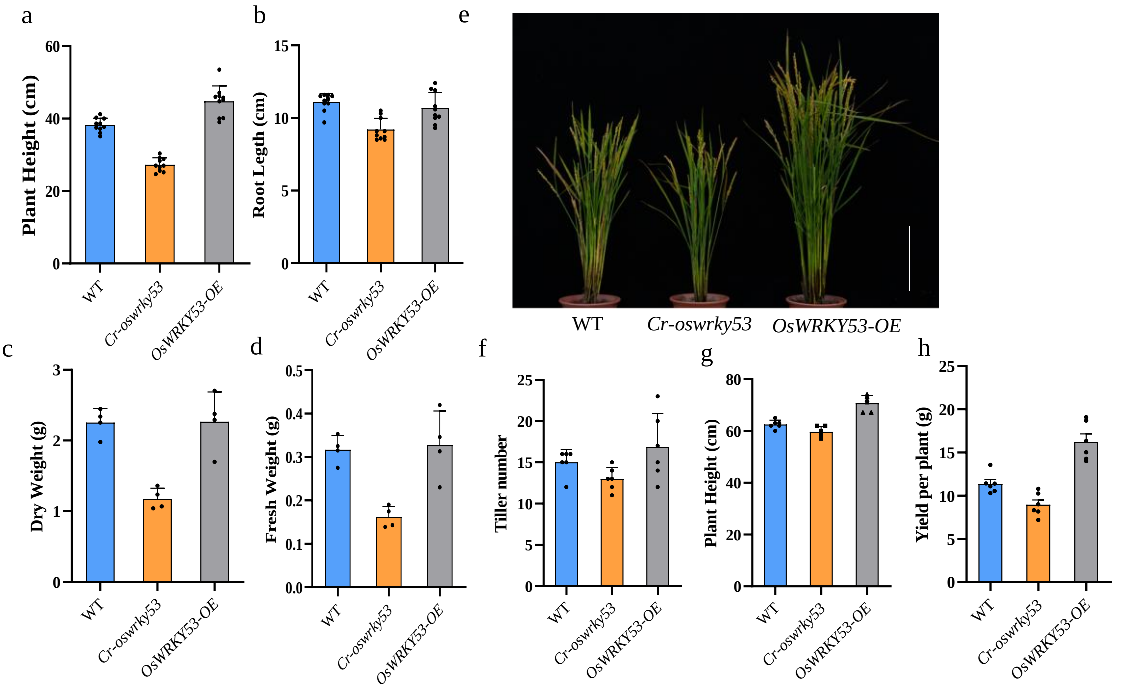

Supplementary Figure 24. Growth characters of *Cr-oswrky53*, *OsWRKY53-OE* and WT. (a-d) Plant height, n = 10 (a), root length, n = 10 (b), dry weight, n = 4 (c) and fresh weight, n = 4 (d) of *Cr-oswrky53*, *OsWRKY53-OE* and WT under hydroponics condition. Twelve seedlings were used to determine dry weight or fresh weight. Four seedlings were grouped randomly to calculate the dry weight or fresh weight shown as one data point. (e) Field phenotypes of *Cr-oswrky53*, *OsWRKY53-OE* and WT. Bar = 18cm. (f-h) Tiller number, n = 6 (f), plant height, n = 6 (g) and yield per plant, n = 6 (h) of *Cr-oswrky53*, *OsWRKY53-OE* and WT. Data were presented as means ± SD. Source data were provided as a Source Data file.

Supplementary Table 1. Phenotypic characteristics of nine salt-related traits under 0 mM NaCl (C) or 140 mM NaCl (S) conditions and nine salt tolerance indices based on the means of the traits in 268 rice accessions.

**Traits**		**Minimum**	**Maximum**	**Mean±SD**	**CV(%)**	**Kurtosis**	**Skewness**
SH (cm)	C	20.30	65.75	38.56+8.05	20.866	-0.183	0.340
	S	9.64	35.74	19.69+4.50	22.866	-0.182	0.487
**ST-SH**	**S/C**	**0.312**	**0.703**	**0.514+0.08**	**14.722**	**-0.252**	**0.151**
SFW(g)	C	0.352	2.693	0.999+0.40	39.841	2.288	1.219
	S	0.104	1.097	0.307+0.12	40.104	6.899	1.798
**ST-SFW**	**S/C**	**0.113**	**0.762**	**0.326+0.11**	**33.254**	**1.093**	**0.762**
SDW(g)	C	0.046	0.385	0.138+0.06	41.589	2.074	1.167
	S	0.040	0.222	0.078+0.03	33.611	4.647	1.660
**ST-SDW**	**S/C**	**0.250**	**1.346**	**0.605+0.17**	**27.553**	**2.966**	**1.159**
SNC (umol/mg)	C	0.032	0.122	0.070+0.02	27.623	-0.554	0.267
	S	0.712	4.265	1.828+0.56	30.400	1.468	0.792
**ST-SNC**	**S/C**	**10.390**	**66.965**	**27.800+11.19**	**40.251**	**1.128**	**1.095**
SKC (umol/mg)	C	0.791	1.520	1.087+0.10	8.841	1.271	0.357
	S	0.379	0.873	0.602+0.08	13.662	0.452	0.097
**ST-SKC**	**S/C**	**0.383**	**0.857**	**0.557+0.08**	**14.698**	**0.239**	**0.377**
SNKR	C	0.028	0.133	0.065+0.02	27.435	0.401	0.495
	S	1.116	7.514	3.103+1.07	34.332	2.132	0.980
**ST-SNKR**	**S/C**	**14.677**	**123.640**	**50.730+20.96**	**41.310**	**1.114**	**1.074**
WC	C	0.833	0.888	0.862+0.01	1.166	-0.157	-0.218
	S	0.546	0.836	0.734+0.06	7.625	0.788	-0.959
**ST-WC**	**S/C**	**0.624**	**0.965**	**0.852+0.07**	**7.662**	**0.765**	**-0.951**
**SR**	**S/C**	**0.000**	**1.000**	**0.541+0.27**	**49.882**	**-1.017**	**-0.204**

SD standard deviation, CV coefficient of variation, SH shoot height, SFW shoot fresh weight, SDW shoot dry weight, SNC shoot Na^+ content, SKC shoot K^+ content, SNKR shoot Na^+/K^+ content ratio, WC water content, SR survival rate, Salt tolerance (ST) was defined as the ratio of salt-related traits under salt treat (S) condition and control (C).

Supplementary Table 2. Pearson's correlation matrix obtained on salt tolerance related traits.

	SR	ST-WC	ST-SH	ST-SFW	ST-SDW	ST-SNC	ST-SNKR	ST-SKC
SR	1							
ST-WC	0.683**	1						
ST-SH	0.163**	0.142*	1					
ST-SFW	0.518**	0.599**	0.487**	1				
ST-SDW	0.122*	0.053	0.473**	0.796**	1			
ST-SNC	-0.347**	-0.433**	-0.192**	-0.514**	-0.371**	1		
ST-SNKR	-0.262**	-0.325**	-0.228**	-0.434**	-0.334**	0.918**	1	
ST-SKC	-0.189**	-0.204**	0.111	-0.169**	-0.089	0.112	-0.256**	1

(**: $P< 0.01$; *: $P< 0.05$, Tukey's test)

Supplementary Table 3. Number of effects by type.

Type	Count	Percent
3_prime_UTR_variant	86,389	0.87%
5_prime_UTR_premature_start_codon_gain_variant	7,570	0.08%
5_prime_UTR_variant	48,423	0.49%
downstream_gene_variant	3,576,197	35.88%
initiator_codon_variant	17	0%
initiator_codon_variant+non_canonical_start_codon	25	0%
intergenic_region	1,962,373	19.69%
intron_variant	362,290	3.64%
missense_variant	81,517	0.82%
missense_variant+splice_region_variant	745	0.01%
non_coding_exon_variant	74,476	0.75%
splice_acceptor_variant+intron_variant	287	0.00%
splice_donor_variant+intron_variant	321	0.00%
splice_region_variant	1,226	0.01%
splice_region_variant+initiator_codon_variant+non_canonical_start_codon	1	0%
splice_region_variant+intron_variant	7,950	0.08%
splice_region_variant+non_coding_exon_variant	1,569	0.02%
splice_region_variant+stop_retained_variant	25	0%
splice_region_variant+synonymous_variant	1,053	0.01%
start_lost	181	0.00%
start_lost+splice_region_variant	2	0%
stop_gained	1,285	0.01%
stop_gained+splice_region_variant	19	0%
stop_lost	273	0.00%
stop_lost+splice_region_variant	59	0.00%
stop_retained_variant	187	0.00%
synonymous_variant	89,539	0.90%
upstream_gene_variant	3,664,029	36.76%

Supplementary Table 4. Detailed information on the DNA polymorphisms assigned to missense-variant in the candidate region on chromosome 1 close to *SKC1*. The candidate genes significantly associated with phenotypic variation were highlighted in green.

Chr	Position (bp)	Ref	Alt	Gene ID	Ref.aa	Alt.aa	Annotation
1	11364629	C	T	*LOC_Os01g19990*	Val	Ile	expressed protein
1	11365222	G	C		Asp	Glu	
1	11367063	C	T	*LOC_Os01g20000*	Gly	Ser	expressed protein
1	11412878	A	G	*LOC_Os01g20110*	Asn	Asp	expressed protein
1	11419649	T	G		Val	Gly	
1	11427028	C	T	*LOC_Os01g20120*	Asp	Asn	expressed protein
1	11427273	T	G		Lys	Gln	
1	11430776	T	G		Asn	His	
1	11462093	G	C	*LOC_Os01g20160*	Leu	Val	OsHKT1;5 - Na+ transporter, expressed
1	11462282	G	C		His	Asp	

Supplementary Table 5. Detailed information on the DNA polymorphisms assigned to missense-variant in the candidate region on chromosome 3 close to *OsHAP2E*. The candidate genes significantly associated with phenotypic variation were highlighted in green.

Chr	Position (bp)	Ref	Alt	Gene ID	Ref.aa	Alt.aa	Annotation
3	16699913	A	G	*LOC_Os03g29350*	Ile	Thr	domain containing protein, expressed
3	16700951	G	C		Ala	Gly	
3	16749510	C	G	*LOC_Os03g29410*	Cys	Ser	tyrosine protein kinase, putative, expressed
3	16749678	C	T		Arg	Lys	
3	16749801	T	C		Lys	Arg	
3	16749802	T	G		Lys	Gln	
3	16749840	A	G		Phe	Ser	
3	16750223	C	T		Val	Ile	
3	16787498	A	G	*LOC_Os03g29460*	Thr	Ala	60S ribosomal protein, putative, expressed
3	16787517	C	T		Ala	Val	
3	16837736	A	G	*LOC_Os03g29540*	Val	Ala	Conserved hypothetical protein
3	16837841	C	G		Ser	Thr	
3	16857608	T	C	*LOC_Os03g29570*	Met	Val	mps one binder kinase activator-like 1A, putative, expressed
3	16880655	G	A	*LOC_Os03g29614*	Pro	Ser	myb-like DNA-binding domain containing protein, putative, expressed
3	16880658	C	T		Gly	Arg	
3	16880660	T	C		His	Arg	
3	16880669	C	T		Gly	Glu	
3	16880702	G	A		Pro	Leu	
3	16937858	A	G	*LOC_Os03g29730*	Val	Ala	Hypothetical protein
3	16967136	A	G	*LOC_Os03g29760*	Asp	Gly	nuclear transcription factor Y subunit, putative, expressed
3	16967165	G	C		Glu	Gln	
3	16969978	A	G		His	Arg	
3	16970121	G	C		Gly	Arg	
3	16995642	G	T	*LOC_Os03g29810*	Ala	Ser	Putative Clp protease homologue, expressed
3	16999296	C	T	*LOC_Os03g29830*	Ala	Val	expressed protein

Supplementary reference

1. Wang, J. *et al*. A DNA methylation reader-chaperone regulator-transcription factor complex activates *OsHKT1;5* expression during salinity stress. *Plant Cell* **32**, 3535-3558 (2020).
2. Xiao, J. *et al*. Rice WRKY13 regulates cross talk between abiotic and biotic stress signaling pathways by selective binding to different cis-elements. *Plant Physiology* **163**, 1868 (2013).
3. Morita, S. *et al*. *In planta* evidence that the HAK transporter OsHAK2 is involved in Na+ transport in rice. *Biosci Biotech Bioch* **87**, 482-490 (2023).
4. Alam, M.M. *et al*. Overexpression of a rice heme activator protein gene (*OsHAP2E*) confers resistance to pathogens, salinity and drought, and increases photosynthesis and tiller number. *Plant Biotechnology Journal* **13**, 85 (2015).
5. Gigyeong, P., Jongjin, P., Jinmi, Y., Yu, S.N. & An, G.H. A RING finger E3 ligase gene, *Oryza sativa Delayed Seed Germination 1* (*OsDSG1*), controls seed germination and stress responses in rice. *Plant Molecular Biology* **74**, 467-478 (2010).
6. Liu, Z. *et al*. OsMKKK70 regulates grain size and leaf angle in rice through the OsMKK4-OsMAPK6-OsWRKY53 signaling pathway. *J Integr Plant Biol* **63**, 2043-2057 (2021).
7. Tian, X. *et al*. Transcription factor OsWRKY53 positively regulates brassinosteroid signaling and plant architecture. *Plant Physiology* **175**, 1337–1349 (2017).
8. Chen, Z. *et al*. Mn tolerance in rice is mediated by MTP8.1, a member of the cation diffusion facilitator family. *Journal of Experimental Botany* **64**, 4375-4387 (2013).
9. Wang, S. *et al*. Phosphorylation and ubiquitination of OsWRKY31 are integral to OsMKK10-2-mediated defense responses in rice. *Plant Cell* **35**, 2391–2412 (2023).
