# Supplementary material for: Genome-wide association studies identify OsWRKY53 as a key regulator of salt tolerance in rice
Source: Nat Commun. 2023 Jun 15;14:3550. doi: 10.1038/s41467-023-39167-0 (PMC10272163; doi:10.1038/s41467-023-39167-0)
Supplement: Supplementary file 2 — Description of Additional Supplementary Files [file 41467_2023_39167_MOESM2_ESM.pdf]

File Name: Supplementary Data 1
Description: ST-related traits in GWAS population with 268 accessions.

File Name: Supplementary Data 2
Description: Rice materials and their membership probabilities corresponding to each subpopulation.

File Name: Supplementary Data 3
Description: List of 83 unique QTLs with 19 loci associated with salt tolerance are highlighted in yellow.

File Name: Supplementary Data 4
Description: Detailed information on the DNA polymorphisms assigned to missense-variant in the candidate region on chromosome 1 close to *HKT2;3*. The candidate genes significantly associated with phenotypic variation are highlighted in green.

File Name: Supplementary Data 5
Description: Detailed information on the DNA polymorphisms assigned to missense-variant in the candidate region on chromosome 1 close to *OsHAK2* and *OsHAK5*. The candidate genes significantly associated with phenotypic variation are highlighted in green.

File Name: Supplementary Data 6
Description: Detailed information on the DNA polymorphisms assigned to missense-variant in the candidate region on chromosome 1 close to *OsWRKY13*. The candidate genes significantly associated with phenotypic variation are highlighted in green.

File Name: Supplementary Data 7
Description: Detailed information on the DNA polymorphisms assigned to missense-variant in the candidate region on chromosome 9 close to *OsDSG1*. The candidate genes significantly associated with phenotypic variation are highlighted in green.

File Name: Supplementary Data 8
Description: Detailed information on the DNA polymorphisms assigned to missense-variant in the candidate region on chromosome 8. The candidate genes significantly associated with phenotypic variation are highlighted in green.

File Name: Supplementary Data 9
Description: Detailed information on the DNA polymorphisms assigned to missense-variant in the candidate region on chromosome 3. The candidate genes significantly associated with phenotypic variation are highlighted in green.

File Name: Supplementary Data 10
Description: Primers in this study.
