# Supplementary material for: Genome-wide association studies identify OsWRKY53 as a key regulator of salt tolerance in rice
Source: Nat Commun. 2023 Jun 15;14:3550. doi: 10.1038/s41467-023-39167-0 (PMC10272163; doi:10.1038/s41467-023-39167-0)
Supplement: Supplementary file 13 — Reporting Summary [file 41467_2023_39167_MOESM13_ESM.pdf]

nature portfolio

Corresponding author(s): Jianmin Wan, Chunming Wang

Last updated by author(s): May 25, 2023

Reporting Summary

Nature Portfolio wishes to improve the reproducibility of the work that we publish. This form provides structure for consistency and transparency in reporting. For further information on Nature Portfolio policies, see our Editorial Policies and the Editorial Policy Checklist.

Statistics

For all statistical analyses, confirm that the following items are present in the figure legend, table legend, main text, or Methods section.

n/a	Confirmed	
☐	☒	The exact sample size (*n*) for each experimental group/condition, given as a discrete number and unit of measurement
☐	☒	A statement on whether measurements were taken from distinct samples or whether the same sample was measured repeatedly
☐	☒	The statistical test(s) used AND whether they are one- or two-sided<br>*Only common tests should be described solely by name; describe more complex techniques in the Methods section.*
☐	☒	A description of all covariates tested
☒	☐	A description of any assumptions or corrections, such as tests of normality and adjustment for multiple comparisons
☐	☒	A full description of the statistical parameters including central tendency (e.g. means) or other basic estimates (e.g. regression coefficient) AND variation (e.g. standard deviation) or associated estimates of uncertainty (e.g. confidence intervals)
☐	☒	For null hypothesis testing, the test statistic (e.g. *F*, *t*, *r*) with confidence intervals, effect sizes, degrees of freedom and *P* value noted<br>*Give P values as exact values whenever suitable.*
☒	☐	For Bayesian analysis, information on the choice of priors and Markov chain Monte Carlo settings
☒	☐	For hierarchical and complex designs, identification of the appropriate level for tests and full reporting of outcomes
☐	☒	Estimates of effect sizes (e.g. Cohen's *d*, Pearson's *r*), indicating how they were calculated

Our web collection on statistics for biologists contains articles on many of the points above.

Software and code

Policy information about availability of computer code

Data collection	Sequencing reads were undertaken with the Hiseq-2500 sequencer (GBS and RNA-seq data); 3000 Rice Genomes Project (genomic data of Rice 3k population).
Data analysis	Tassel 5.2.13 ,Bowtie2, SPSS v18.0 statistical softwares were used for statistical analysis; Microsoft Excel 2020; GraphPad Prism v8.0.1 were used for graph drawing, and SPSS v17.0 was used for significance analysis (two-sided Student's t test, Tukey's test).

For manuscripts utilizing custom algorithms or software that are central to the research but not yet described in published literature, software must be made available to editors and reviewers. We strongly encourage code deposition in a community repository (e.g. GitHub). See the Nature Portfolio guidelines for submitting code & software for further information.

Data

Policy information about availability of data

All manuscripts must include a data availability statement. This statement should provide the following information, where applicable:

- Accession codes, unique identifiers, or web links for publicly available datasets
- A description of any restrictions on data availability
- For clinical datasets or third party data, please ensure that the statement adheres to our policy

The GBS data and RNA-seq data has been upload to the website NCBI SRA (Sequence Read Archive) database, accession number: SRR13518263, SRR13493593,

SRR13493703, SRR13495248. Rice3K genomic SNPs can be down loaded from the database (http://snp-seek.irri.org/) and the data of the 42 wild rice genomic SNPs used in the haplotype network analysis can be down loaded from NCBI (https://www.ncbi.nlm.nih.gov/bioproject/PRJNA4078 20).

Research involving human participants, their data, or biological material

Policy information about studies with human participants or human data. See also policy information about sex, gender (identity/presentation), and sexual orientation and race, ethnicity and racism.

Reporting on sex and gender	Not applicable
Reporting on race, ethnicity, or other socially relevant groupings	Not applicable
Population characteristics	Not applicable
Recruitment	Not applicable
Ethics oversight	Not applicable

Note that full information on the approval of the study protocol must also be provided in the manuscript.

Field-specific reporting

Please select the one below that is the best fit for your research. If you are not sure, read the appropriate sections before making your selection.

☒ Life sciences ☐ Behavioural & social sciences ☐ Ecological, evolutionary & environmental sciences

For a reference copy of the document with all sections, see nature.com/documents/nr-reporting-summary-flat.pdf

Life sciences study design

All studies must disclose on these points even when the disclosure is negative.

Sample size	We gathered the various rice accessions, including 100 indica, 50 aus and 118 japonica which were originating from around the world, for phenotypic identification under salt stress.
Data exclusions	No data excluded.
Replication	All experiments in this study were repeated independently at three times. For qRT-PCR three biologically independent samples were used each time. For Na+ flux analysis, dual–luciferase assay and salt tolerance identification, the results representative of three independent experiments. All attempts at replication were successful for all experiments.
Randomization	All the samples were arranged randomly into experimental groups.
Blinding	The investigators were blinded to group allocation during data collection. Two investigators participated in experiment design and conduction. Samples were randomly numbered by one investigator, and the other investigator was responsible for data collection and analysis.

Reporting for specific materials, systems and methods

We require information from authors about some types of materials, experimental systems and methods used in many studies. Here, indicate whether each material, system or method listed is relevant to your study. If you are not sure if a list item applies to your research, read the appropriate section before selecting a response.

Materials & experimental systems

n/a	Involved in the study	
☐	☒	Antibodies
☒	☐	Eukaryotic cell lines
☒	☐	Palaeontology and archaeology
☒	☐	Animals and other organisms
☒	☐	Clinical data
☒	☐	Dual use research of concern
☐	☒	Plants

Methods

n/a	Involved in the study	
☒	☐	ChIP-seq
☒	☐	Flow cytometry
☒	☐	MRI-based neuroimaging

Antibodies

Antibodies used	Anti-OsWRKY53 (Beijing Protein Innovation, AbP80095-A-SE), anti-GST (Beijing Protein Innovation, AbM59001-2H5-PU), anti-MAPK6 (Beijing Protein Innovation, AbP80140-A-SE), anti-phospho-p44/42MAPK (Cell Signaling Technology, ＃9101)
Validation	Anti-OsWRKY53 was used in ChIP-qPCR assay. Anti-phospho-p44/42 MAPK was used in western blot to detect endogenous levels of p44 and p42 MAP Kinase (Erk1 and Erk2) . Anti-MAPK6 and anti-GST were used in western blot.

Dual use research of concern

Policy information about dual use research of concern

Hazards

Could the accidental, deliberate or reckless misuse of agents or technologies generated in the work, or the application of information presented in the manuscript, pose a threat to:

No	Yes	
☒	☐	Public health
☒	☐	National security
☒	☐	Crops and/or livestock
☒	☐	Ecosystems
☒	☐	Any other significant area

Experiments of concern

Does the work involve any of these experiments of concern:

No	Yes	
☒	☐	Demonstrate how to render a vaccine ineffective
☒	☐	Confer resistance to therapeutically useful antibiotics or antiviral agents
☒	☐	Enhance the virulence of a pathogen or render a nonpathogen virulent
☒	☐	Increase transmissibility of a pathogen
☒	☐	Alter the host range of a pathogen
☒	☐	Enable evasion of diagnostic/detection modalities
☒	☐	Enable the weaponization of a biological agent or toxin
☒	☐	Any other potentially harmful combination of experiments and agents
